# Metabolic Profiling of a Porcine Combat Trauma-Injury Model Using NMR and Multi-Mode LC-MS Metabolomics—A Preliminary Study

**DOI:** 10.3390/metabo10090373

**Published:** 2020-09-16

**Authors:** Anna Karen Carrasco Laserna, Yiyang Lai, Guihua Fang, Rajaseger Ganapathy, Mohamed Shirhan Bin Mohamed Atan, Jia Lu, Jian Wu, Mahesh Uttamchandani, Shabbir M. Moochhala, Sam Fong Yau Li

**Affiliations:** 1 Department of Chemistry, Faculty of Science, National University of Singapore, 3 Science Drive 3, Singapore 117543, Singapore; chmakcl@nus.edu.sg (A.K.C.L.); fang_guihua@hsa.gov.sg (G.F.); umahesh@dso.org.sg (M.U.); 2 Defence Medical and Environmental Research Institute, DSO National Laboratories, 27 Medical Drive, Singapore 117510, Singapore; lyiyang@dso.org.sg (Y.L.); grajaseg@dso.org.sg (R.G.); ljia@dso.org.sg (J.L.); wjian@dso.org.sg (J.W.); 3 Forensic Science Division, Health Services Authority, 11 Outram Road, Singapore 169078, Singapore; 4 School of Applied Sciences, Temasek Polytechnic, 21 Tampines Ave 1, Singapore 529757, Singapore; shirhana@tp.edu.sg; 5 Department of Pharmacology, Yong Loo Lin School of Medicine, National University of Singapore, Blk MD3, 16 Medical Drive, Singapore 117600, Singapore; 6 NUS Environmental Research Institute, National University of Singapore, T-Lab Building, 5A Engineering Drive 1, Singapore 117411, Singapore

**Keywords:** trauma, hemorrhagic shock, metabolomics, LC-MS, NMR

## Abstract

Profiles of combat injuries worldwide have shown that penetrating trauma is one of the most common injuries sustained during battle. This is usually accompanied by severe bleeding or hemorrhage. If the soldier does not bleed to death, he may eventually succumb to complications arising from trauma hemorrhagic shock (THS). THS occurs when there is a deficiency of oxygen reaching the organs due to excessive blood loss. It can trigger massive metabolic derangements and an overwhelming inflammatory response, which can subsequently lead to the failure of organs and possibly death. A better understanding of the acute metabolic changes occurring after THS can help in the development of interventional strategies, as well as lead to the identification of potential biomarkers for rapid diagnosis of hemorrhagic shock and organ failure. In this preliminary study, a metabolomic approach using the complementary platforms of nuclear magnetic resonance (NMR) spectroscopy and liquid chromatography coupled with mass spectrometry (LC-MS) was used to determine the metabolic changes occurring in a porcine model of combat trauma injury comprising of penetrating trauma to a limb with hemorrhagic shock. Several metabolites associated with the acute-phase reaction, inflammation, energy depletion, oxidative stress, and possible renal dysfunction were identified to be significantly changed after a thirty-minute shock period.

## 1. Introduction

Trauma injury has been identified as one of the leading causes of death worldwide [1]. The lethality of trauma is even higher in combat scenarios wherein the wounding agents are intentionally fatal, and the resources for the administration of treatment are limited compared to civilian settings. Injuries in combat scenarios are mostly due to penetrating trauma from explosions and gunshots [2,3]. Penetrating injuries usually consist of tissue damage, possible bone fracture, and if blood vessels are ruptured, severe bleeding or hemorrhage can also occur. The amount of oxygen reaching the organs can significantly decrease with the loss of blood, leading to a condition known as “shock” [4]. Trauma hemorrhagic shock (THS) triggers a complex cascade of cellular and molecular changes in the body that compromises the inflammatory response. If regulation of the inflammatory response fails, systemic inflammatory response syndrome (SIRS) may develop, affecting organs far from the site of injury. This could then lead to multiple organ dysfunction syndrome (MODS), progress to multiple organ failure (MOF), and possibly death. MOF develops in 10–30% of trauma patients with a subsequent mortality rate of over 30% [5,6]. 

Early detection of the onset of THS is crucial for the implementation of appropriate interventions, especially in combat scenarios wherein the resources and the window for effective pre-hospital care are highly limited. Criteria for the diagnosis of hemorrhagic shock are mainly based on the measurement of blood loss volume and vital signs, such as pulse rate, blood pressure, and respiratory rate [7]. However, monitoring of these vital signs will not be useful in the case of asymptomatic patients wherein compensatory mechanisms can maintain homeostasis, which is particularly common among younger patients [8]. There is a need for more reliable indicators of the onset and progression of THS and a better understanding of the occurring physiological changes.

Metabolomics has been defined as “the comprehensive analysis in which all the metabolites are identified and quantified” [9]. It has been used in different fields of research to explain underlying mechanisms to certain conditions and identify potential biomarkers that can be used for diagnosis. However, its use in the study of critical injuries and related conditions, such as THS, is still relatively new [10]. Most of the reported trauma injury metabolomic studies so far have used ^1^H-NMR [11,12,13,14,15]. While there are advantages to the use of NMR, such as providing a global overview of the metabolic profile, having minimal sample preparation requirements, and being a non-destructive, quantitative, highly robust, and being reproducible technique, it can still be limited in terms of sensitivity. Furthermore, researchers often experience difficulties in identifying metabolites from resonance signals that are highly overlapped. The use of mass spectrometry-based techniques can provide increased sensitivity and metabolite information based on mass and fragmentation data, circumventing these limitations. More recently, trauma hemorrhagic shock and ischemia/reperfusion studies of rat and mouse models have been performed using LC-MS [16,17,18]. While there are advantages to using each of these platforms, none of them can cover the whole metabolome. Thus, to increase the metabolome coverage and attain a clearer picture of the occurring metabolic changes, the use of two or more platforms is needed. 

In this study, NMR and LC-MS were both used to obtain a comprehensive profile of the metabolomic changes occurring in a porcine model of combat trauma. LC-MS analyses were performed using two chromatographic modes, namely hydrophilic interaction (HILIC) and reverse phase (RP) liquid chromatography (LC). RP-LC was optimized for the analysis of non-polar to slightly polar metabolites. In RP-LC, highly polar compounds were not retained and thus eluted at the void volume. HILIC-LC was optimized for the analysis of polar and hydrophilic metabolites. For each chromatographic mode, analyses were further carried out in both positive and negative ionization modes, resulting in a multi-mode LC-MS metabolomics approach to expand the metabolome coverage.

## 2. Results

### 2.1. Mortality, Injury Severity, and Blood Chemistry

Samples were obtained from pigs subjected to either sham or trauma injury at baseline and 30 minutes after the injury phase. Blood oxygen saturation (sO_2_) level was at 100% in sham and traumatized animals as a result of oxygen supplementation during anesthesia. Initial profiling was done using ^1^H-NMR with samples taken from five pigs subjected to trauma injury and five pigs subjected to a sham procedure. However, one of the sham pigs developed elevated body temperature (beyond 38 degrees Celsius) for a prolonged period during the experiment, suggestive of an underlying infection. To avoid confounding outcomes, samples from this animal were removed from the study. LC-MS analyses tend to have higher instrument variability as compared to ^1^H-NMR analysis. To obtain a more reliable LC-MS profile of the trauma group, the sample size of the trauma group for the LC-MS analyses was increased by including samples from five additional pigs subjected to the same protocol up to the injury phase but were still used for further resuscitation studies. The mean survival duration for the trauma animals used for the NMR and LC-MS analyses was 19.1 ± 5.6 and 17.8 ± 5.1 h (*p* < 0.05), respectively. Sham animals were euthanized with an overdose injection of sodium pentobarbital after 48 h of continuous monitoring. Following a 30-minute shock induction period, as described in Wong et al. [19], core temperature, blood pH, and pressure declined to sub-physiologic levels in the trauma animals. Severe blood loss, as evidenced by the low hematocrit levels, coupled with exogenous cooling provided by the cooling vest, caused the animals’ body temperature to drop (Table 1). Massive blood loss was also responsible for the hypotension reported in these highly anemic animals. In addition, deranged blood pH, base excess, and lactate values were consistent with the observation of an acidotic state, reported herein. The rise in blood sodium and chloride levels was attributed to the large volume of saline introduced during the resuscitation of the trauma animals.

### 2.2. ^1^H-NMR Analysis

**Figure 1:**
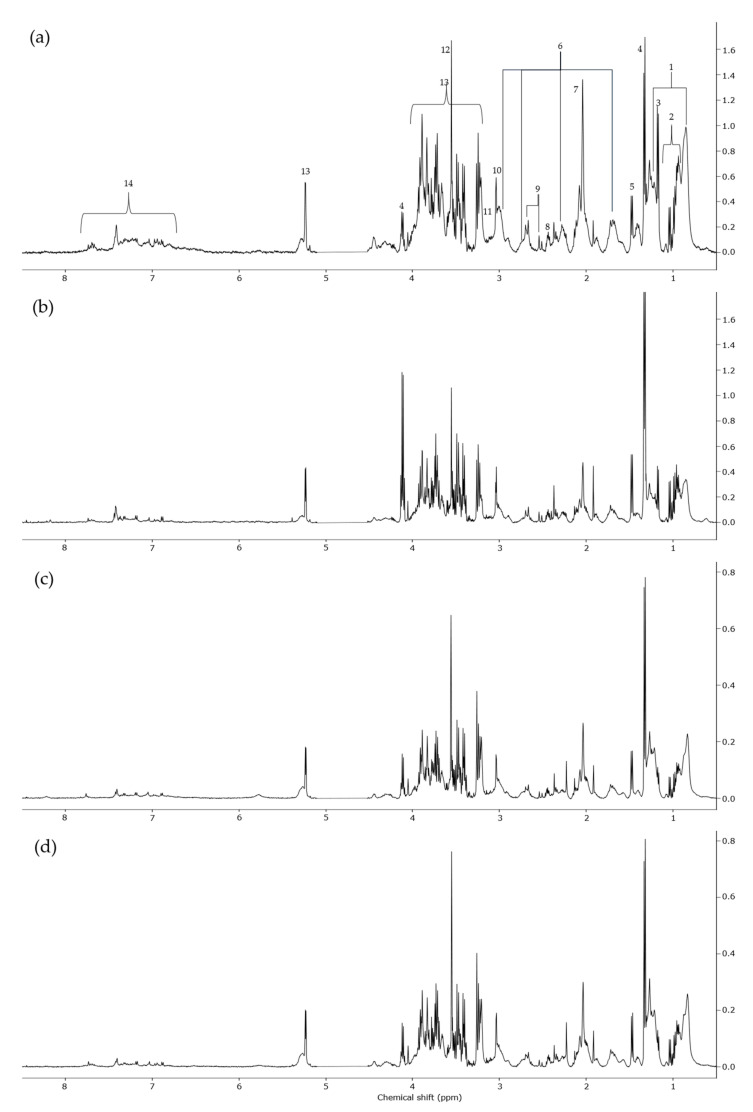
Comparison of the NMR spectra of plasma samples from pigs before and thirty minutes after the trauma injury or sham procedures: (**a**) before trauma; (**b**) after trauma; (**c**) before sham, and (**d**) after sham procedure. Peak assignments of some of the identified metabolite: (1) Low-density and very-low-density lipoproteins (LDLs and VLDLs), (2) isoleucine, leucine, valine, (3) 3-hydroxybutyrate, (4) lactate, (5) alanine, (6) lipids, (7) *N*-acetylglycoproteins (*N*-AG), (8) glutamine, (9) citrate, (10) creatinine, (11) albumin (lysyl), (12) glycine, (13) glucose, (14) phenylalanine, tyrosine, tryptophan, and other aromatic metabolites.

Proton NMR analysis was performed to obtain a global overview of the metabolic changes in the plasma of pigs after combat trauma injury. Figure 1 shows a comparison of representative spectra for sham and combat trauma samples. The spectral profiles of the sham samples are very similar, whereas there are observable differences in the profile of the pig subjected to trauma injury before and after the injury phase. The main differences are the reduction in the intensity of peaks in the region of 0.5–1.3 ppm and 1.9–2.1 ppm. Resonances in this region are mainly from low- and very low-density lipoproteins (LDLs and VLDLs) and glycoproteins [20]. Principal component analysis (PCA) was used to visualize the inherent groupings or clustering in the binned NMR data. 

**Figure 2:**
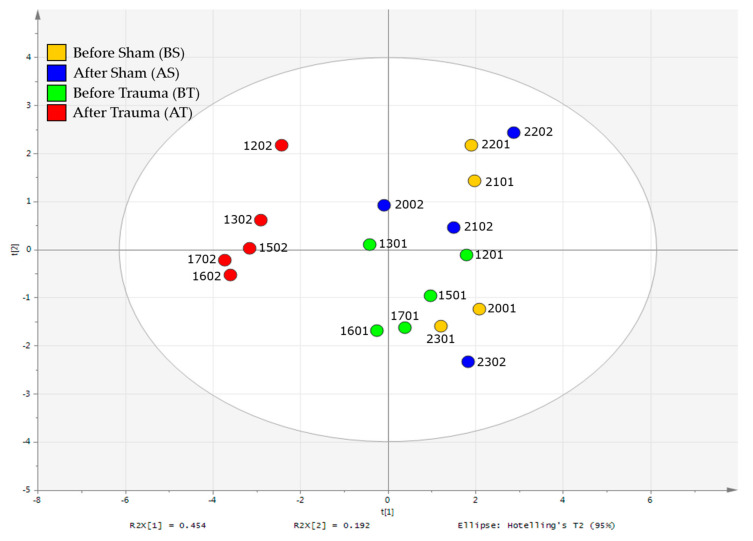
Principal component analysis (PCA) score plot of the untargeted binning NMR data. Samples from pigs before and after the sham procedure are colored yellow and blue, respectively. Samples from pigs before and after trauma injury are colored green (BT) and red (AT). For the sample IDs, the first two digits denote the pig ID followed by the timepoint at which the blood was drawn: (01)—before trauma/sham and (02)—after trauma/sham. Model variation (R2X) represents the fraction/ percentage of the variation of the model X explained by component [i].

Figure 2 shows the score plot for the first two components of the PCA model, with the first and second components accounting for 45.4% and 19.2% of the variation of the model (R2X). The overlapped clustering of the before trauma (BT) and before/after sham (BS/AS) samples, with the marked separation of the after trauma (AT) samples, is an indication that the differentiation observed can be mainly attributed to the metabolic changes resulting from the induced trauma injury and that the surgical procedures have less contribution to the metabolic variations.

**Figure 3:**
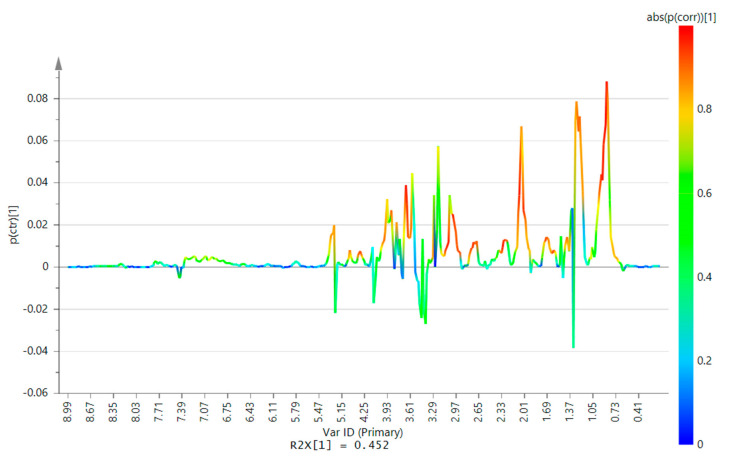
Orthogonal projection to latent structures-discriminant analysis (OPLS-DA) S-line plot of the AT samples versus the sham and BT samples. An increasingly red color of the spectral line indicates higher importance to the differentiation of the AT samples from the rest. Positive y-axis values indicate decreased levels after injury of associated features after trauma, while negative values indicate increased levels after injury. Features differentiating AT samples: (1) cholesterol, (2) LDLs and VLDLs, (3) acetylglycoproteins (N-AG), (4) albumin (lysyl), (5) lipids, (6) SN-glycerophosphocholine (SN-GPC), (7) unsaturated lipids, (8) lactate, (9) glucose.

Supervised analysis using orthogonal projection to latent structures-discriminant analysis (OPLS-DA) was performed to identify the spectral features that differentiate the AT samples from the BT and sham samples. The use of OPLS-DA allows the variation that is unrelated to the differentiation of the AT samples to be separated into orthogonal components, thereby facilitating easier identification of variables that are truly contributing to the differentiation. OPLS-DA has a feature called the S-line plot that can be used to visualize differentiating features in NMR metabolomics data with a 2-group comparison. In this case, one group is the combination of BT, BS, and AS samples, while the other group is the AT samples. The plot (Figure 3) is similar to an NMR spectrum, with the chemical shift as the horizontal axis. The y-axis is the predictive loadings showing the influence of the variables on the differentiation. Those with positive values are those higher in sham and baseline, whereas those with negative y-axis values are those higher after trauma. The color indicates the degree of the influence on the differentiation. Those with colors in the red region of the spectrum are the ones that are strongly influencing. The compounds, or group of compounds, corresponding to the signal were identified based on reported literature [20] and from the Chenomx Profiler. 

Visual inspection revealed that AT samples were distinct from the other samples due to the marked decrease in the levels of LDLs, VLDLs, and glycoproteins. From the S-line plot, other metabolites were also found to be strongly differentiating, such as the lipid signals, cholesterol, SN-glycero-3-phosphocholine (SN-GPC), and albumin. Lactate and glucose levels were raised following trauma, but its contribution to the differentiation was not strong, as shown by the color of the spectral line, possibly due to high variations in levels of glucose and lactate across the samples. The significant features based on OPLS-DA VIP were then taken, and Wilcoxon–Mann–Whitney test was performed on their fold change values to identify the variables with a significant difference in fold changes between the sham and trauma samples. The molecules associated with the signal in terms of the chemical shift and multiplicities were identified (Supplementary Materials, Table S1). Mainly, the same groups of molecules identified from the S-plot were identified to be the significant features. However, for some variables, there was a high degree of overlap among different metabolites and the lipid and protein signals, making it difficult to pinpoint which molecule was contributing toward the differentiation. The spectra were further profiled using Chenomx Profiler to determine the concentrations of the metabolites, particularly those that overlapped with the protein and lipid signals (Supplementary Materials, Table S2). Fold changes among the profiled metabolites were found to be significantly different between the sham and trauma samples only for choline. This confirms that for the remaining regions with high signal overlap, it is the signal from the lipids and proteins and not those of the overlapped metabolites that contribute towards the differentiation of the samples.

### 2.3. HILIC and RP-LC-MS Analysis

Due to the relatively lower sensitivity of the NMR and the difficulty in identifying metabolites in regions of high signal overlap, LC-MS was also employed to extend the metabolome coverage assessed in this study further. Comprehensive coverage was obtained by using two chromatographic separation modes, HILIC and RP-LC, and the use of both positive and negative electrospray ionization. By using both separation modes, the range of metabolites analyzed encompassed low to high polarity metabolites. The use of the same gradient program for the positive and negative MS acquisition in each of the separation modes also allowed metabolites that can ionize in both positive and negatively charged forms to elute at the same retention time, providing further confirmation of the metabolite identification. Representative LC-MS base peak chromatograms (BPC) of samples before and after combat trauma or sham are available in the Supplementary Materials (Figures S1 and S2). It can be seen across all LC-MS analysis modes that several chromatographic features were altered when comparing BT and AT samples, whereas BS and AS samples looked similar. 

**Figure 4:**
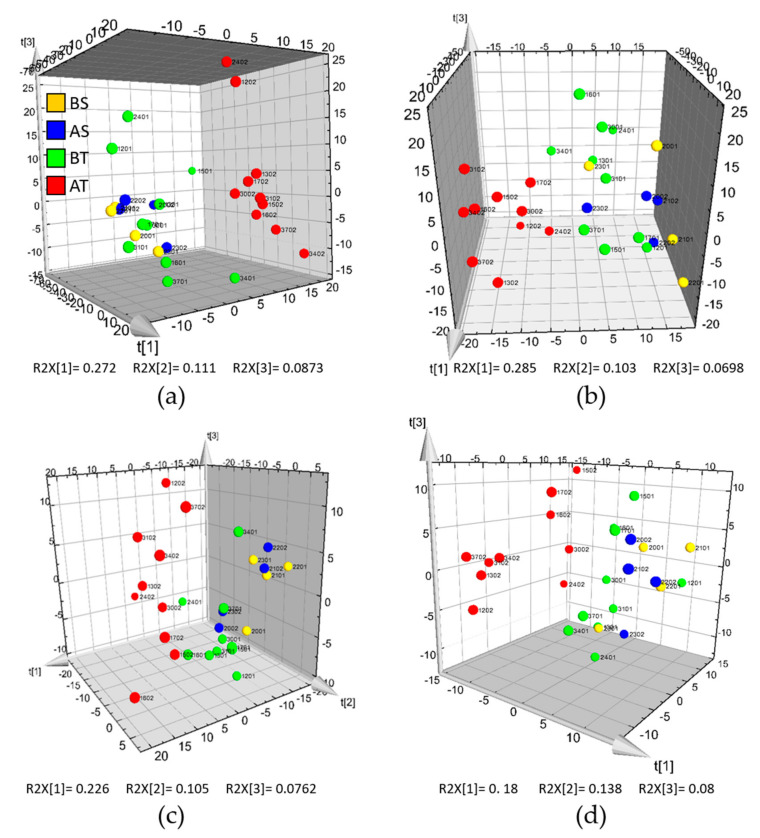
3D PCA score plots of the hydrophilic interaction (HILIC) and reverse phase (RP) LC-MS datasets: (**a**) HILIC LC-MS in positive ionization mode; (**b**) HILIC LC-MS in negative ionization mode; (**c**) RP LC-MS in positive ionization mode; and (**d**) RP LC-MS in negative ionization mode. Samples from pigs before and after the sham procedure are colored yellow (BS) and blue (AS), while those before and after trauma injury are red (BT) and green (AT), respectively. For the sample IDs, the first two digits denote the pig ID, followed by the time point at which the blood was drawn: (01)—before trauma/sham and (02)—after trauma/sham.

PCA was performed on the LC-MS datasets to identify groupings and trends, as well as to detect the presence of outliers. Figure 4 shows that there was a distinct clustering of the AT samples, while the sham and baseline samples were clustered together, similar to the NMR results. 3D score plots were used for visualization to see more clearly the differentiation of the AT samples. There were more variables in the LC-MS datasets, particularly for the HILIC-LC-MS datasets, as compared to the NMR dataset, and these contributed to the variance of the models regardless of association with AT samples. Since unrelated variance did affect the first two principal components of the models, the third component was used for better visualization of the clustering. 

All the LC-MS analyses were done with a pooled quality control (QC) sample for each batch of 10 samples and with randomization of the order of analysis. Shewhart control charts prepared for each dataset ensured QC samples were within the critical limits (Supplementary Materials, Figure S3). 

OPLS-DA was again used to identify features significant to the differentiation of the AT samples. VIP scores of analyzed samples were taken, and those with VIP > 1 and cvSE < VIP were considered to be significant. Hundreds of variables met the said criteria for each dataset. The HILIC technique generated 389 and 498 significant variables for positive and negative modes, respectively, whereas the RPLC datasets had 185 and 219 variables correspondingly. Fold change values of the short-listed variables were subjected to Wilcoxon–Mann–Whitney test with Benjamini–Hochberg false discovery rate (FDR) to further narrow down the list of variables. From the narrowed-down features, corresponding metabolites were identified based on accurate mass and MS/MS data. Table 2 illustrates some metabolites identified to be significantly differentiating using LC-MS belonging to certain metabolite classes found to be highly represented among the differentiating metabolites (for the full list, see Supplementary Materials, Table S3). It can be observed that while there were some metabolites identified from multiple LC-MS modes (HILIC or RP, positive or negative ionization), most of them were only found in one mode. This shows that the use of complementary chromatographic separations, such as HILIC and RP, as well as both positive and negative MS ionization, is needed to attain a comprehensive metabolic profile. 

A large proportion of the identified metabolites belong to the classes of glycerophospholipids and sphingolipids, which were mostly reduced after injury. There was also a significant decrease in SN-GPC and a corresponding significant increase in choline, similar to what has been observed from our NMR data. Taken together, this indicates a perturbation in lipid metabolism. Other metabolites identified included purine and pyrimidine bases, as well as amino acids and their derivatives, which were observed to be significantly higher than baseline levels. A concomitant increase in levels of some acylglycines and glutarylcarnitine was also observed in AT samples. Accumulation of this group of metabolites has been attributed to disorders of mitochondrial fatty acid β-oxidation [21] and increased catabolism of certain amino acids [22]. Glucuronide metabolites, such as 3-indolecarboxylic acid glucuronide, *p*-cresol glucuronide, and phenethylamine glucuronide, were also determined to be significantly elevated following trauma. 

### 2.4. Relevant Metabolic Pathways 

**Figure 5:**
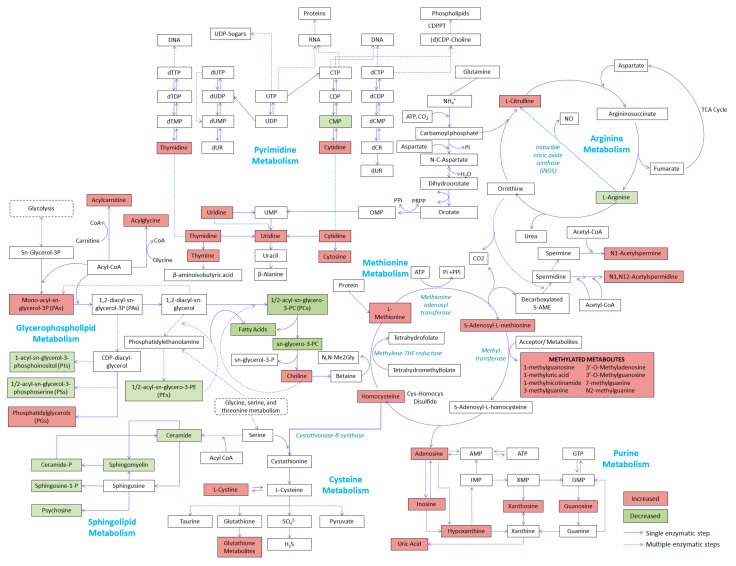
Schematic representation of the top metabolic pathways perturbed after trauma injury. Metabolites in red boxes are those found to have significantly increased after trauma compared to sham, while those in green boxes are those significantly reduced after the injury. Arrows with solid lines represent direct conversions through single enzymatic steps, while those with dashed lines are those requiring multiple enzymatic steps.

A log 2 fold change table of significantly differentiating metabolites from the different datasets was generated and used for pathway analysis using Metaboanalyst, to identify which pathways were affected after the injury (see Supplementary Materials, Table S4). We used the Kyoto Encyclopedia of Genes and Genomes (KEGG) human metabolite library in Metaboanalyst as a reference since a porcine library was unavailable. We have taken into consideration the physiological, hemodynamic, and metabolic similarities [23,24] between porcine and humans in our selection. Represented in Figure 5 are the top pathways identified to be significantly perturbed after injury. Decreased levels of glycerophospholipids and sphingolipids after injury reflect heightened lipolysis and a possible downregulation of their synthesis. Increased catabolism of purine and pyrimidine nucleotides could also be observed with the elevation of several downstream nucleosides and bases. The perturbation of arginine metabolism was also observed with increased conversion of arginine to citrulline. It can also be seen that several metabolites linked to cysteine-methionine metabolism were elevated after the onset of injury, particularly the methylated metabolites, indicating perturbation of the said pathway.

## 3. Discussion

In this study, the metabolic changes occurring in a porcine model of combat trauma injury was studied using NMR and LC-MS as analytical platforms. Pigs are often used in trauma and hemorrhagic shock/resuscitation models due to their similarity in hemodynamic and cardiovascular mechanisms with humans, as well as comparable drug response and metabolism [25], with some metabolites, such as lactate and succinate, having similar levels in pigs post-shock when compared to critically injured patients [26]. Merrifield and co-workers demonstrated that metabolites found in serum, urine, and certain tissues in pigs, are comparable to those found in humans and rodents, with some differences observed in urine due to differences in phase II conjugation. As an example, p-cresol is converted to the glucuronide form in phase II metabolism for pigs, whereas it is mainly converted to the sulfate form in humans [24]. 

The cross-platform approach used in this study has further expanded the metabolome coverage characterizing trauma injury with hemorrhagic shock. Results show agreement with previous studies of trauma injury, in terms of the observed changes in a number of purines and pyrimidines (hypoxanthine, adenosine, choline, cytidine), in glycerophospholipids, as well as in certain amino acids and amino acid derivatives [11,12,27]. The use of both NMR and LC-MS resulted in the identification of more metabolites belonging to these metabolite groups, as well as some additional metabolites and proteins, which has provided a more detailed picture of the affected biological processes.

From the NMR analysis, most of the changes observed after the trauma injury were associated with the acute phase reaction of the inflammatory response. This was mainly indicated by the reduction in plasma protein levels (albumin and N-acetyl glycoproteins), as well as the lipoproteins. While the reduction in protein levels can be attributed to heightened protein catabolism, which normally occurs after injury [28], it can also be due to the effects of inflammatory mediators, such as cytokines, on transcription factors that, in turn, dictate the rate of protein synthesis in the liver. Apoproteins that make up lipoproteins were also identified to be negative acute-phase proteins [29], which means the production of lipoproteins during the acute phase was restricted. Synthesis of albumin can also be inhibited by inflammatory cytokines [30]. *N*-acetyl glycoproteins (*N*-AG) consist of both positive and negative acute-phase proteins. The observed decrease in this study did not correlate with the expected change in N-AG, which is often found to increase in the presence of inflammation and in other disease states [31]. This may be due to a higher rate of protein catabolism and reduced production of negative acute-phase glycoproteins, which results in a net reduction in the overall protein levels [32]. 

Lipid peroxidation due to oxidative stress under ischemic conditions of hemorrhagic shock can also contribute to reduced levels of lipids. Oxidative stress occurs when there is an excess of reactive oxygen species (ROS) over what can be controlled by the body’s antioxidant defense mechanism [33]. Damage-associated molecular patterns (DAMPs), such as High Mobility Group Box-1 (HMGB-1), can be released from dying cells or immune cells during hemorrhagic shock, which can bind to receptors and trigger excessive production of ROS by certain oxidative enzymes, such as xanthine oxidase [34]. Incidentally, we previously reported an elevated state of HMGB-1 in the same poly-traumatized porcine model [19], and this observation corroborated with our metabolomics outcomes, where a high level of circulating HMGB-1 could be responsible for the overproduction of metabolites, such as hypoxanthine and xanthosine, observed after injury. Another explanation for lower levels of lipids observed could be attributed to heightened lipolysis, evidenced by the concomitant reduction in glycerophospholipids and SN-GPC, together with the concurrent increment of choline levels. Increased conversion of arginine to citrulline involving the inducible nitric oxide synthase (iNOS) enzyme, leads to the production of peroxynitrite molecules that can also cause lipid peroxidation.

In this study, increased levels of several purine metabolites were observed after the trauma injury, indicating increased dephosphorylation of the high energy nucleotides, adenosine triphosphate (ATP), and guanosine triphosphate (GTP). Accumulation of purine metabolites after hemorrhagic shock has been previously reported and was associated with cellular damage after shock in the porcine model of blunt trauma/hemorrhagic shock by Lexcen and co-workers [11]. Hypoxanthine, xanthine, and uridine have been previously proposed as indicators of ATP depletion [35]. Pyrimidine nucleosides and some of the bases were also observed to be elevated in the plasma after injury, together with a significant decrease in cytidine monophosphate (CMP). As with their purine counterparts, pyrimidine nucleotides are not only used for nucleic acid synthesis, but they can also be used for energy production via the release of high energy phosphates. However, they are used to a lesser extent as compared to ATP.

Several methylated purine metabolites, polyamines, l-cystine, *S*-adenosylmethionine (*S*-AME), and glutathione metabolites were significantly increased after the injury. This demonstrates that the cysteine–methionine pathway, particularly the *S*-AME cycle, is perturbed after THS, possibly in defense against heightened inflammatory response and oxidative stress. Cysteine–methionine metabolism is highly important in the inflammatory response. Besides the modulation of cytokines, it is the source material of endogenous glutathione production, one of the main endogenous antioxidants needed in the defense against oxidative stress [36]. In methionine metabolism, l-methionine is converted to *S*-AME, which plays an important role in the trans-methylation and trans-sulfuration reactions, as well as in the synthesis of polyamines. *S*-AME can regulate the re-methylation of the toxic homocysteine and favor the production of glutathione. It has also been reported that *S*-AME can modulate TNF-α and IL-10 [37], thus also contributing to the regulation of the inflammatory response. 

Glucuronides are formed through the conjugation of the hydrophilic glucuronide moiety from UDP-glucuronic acid to a compound, making it less toxic and more water-soluble for excretion. Accumulation of glucuronides in plasma could indicate renal or hepatic dysfunction. One of the glucuronides found to be elevated in the AT samples is *p*-cresol glucuronide. Its primary metabolite, *p*-cresol, is a toxin that grows favorably in the intestine through the action of certain bacteria under uremic conditions [38]. Uremia or uremic syndrome is a serious complication of renal failure wherein urea, and other compounds that should be metabolized by the kidney and/or excreted in the urine accumulate in the blood [39]. Aside from p-cresol glucuronide, phenethylamine glucuronide, and some other metabolites found to be elevated after THS are also classified to be uremic retention solutes or uremic toxins by the European Uremia Toxin (EUTox) Work Group [40] (Table 2). The elevation of these uremic metabolites after THS can be an indication of acute renal dysfunction due to the ischemia resulting from hemorrhagic shock. Uremia has traditionally been more associated with chronic kidney disease (CKD), whereas the study of its association with acute kidney injury (AKI) is still at an early stage [41]. Kidney injury is a common complication arising from ischemia [42]. It has been shown in an in vitro model that ATP depletion can cause apoptosis or necrosis [43]. Mice and rat models of ischemic injury have also shown a protective effect of guanosine supplementation against renal failure [44]. The depletion of ATP and GTP after the trauma injury, as evidenced by the elevation of the purine bases, may contribute to the development of renal dysfunction in this THS model. 

Lusczek and co-workers reported a few metabolites found to be potential markers of injury (hypoxanthine and 5-aminolevulinate), injury severity (succinate and hypoxanthine), and mortality (succinate and malonate) in combat trauma patients [15]. Other trauma metabolomic studies also highlighted succinate as a predictor of mortality [45], with glycolysis and the tricarboxylic acid (TCA) metabolism found to be perturbed in trauma with hemorrhagic shock [46]. Interestingly, we did not find metabolites of glycolysis and the tricarboxylic acid cycle to be particularly differentiating in this porcine model. Rather, other metabolites and pathways were found to be more differentiating. This might be due to differences in the injury models used, particularly with the higher severity of hemorrhage used in this model (60% blood loss). In addition, the inducement of hypothermia before shock might also influence the glycolytic and TCA pathways, thereby resulting in the said pathways not being significantly differentiating in this model. Decreased muscle metabolism has been reported with induced hypothermia during resuscitation [13]. However, the effect of hypothermia on metabolism prior/during shock is not yet well-established. This will have to be evaluated through further experiments with this model. Meanwhile, the results of this study support the report of Clendenen and co-workers, wherein the combination of tissue and hemorrhagic shock mainly affects nitrogen metabolism [47].

This study also has some limitations. The number of pigs used in this preliminary study was relatively small. A better model and representation of the metabolic profiles will be attained with a bigger sample size. In addition, only male pigs were used for experimentation. The intent was to reflect the majority of the injured soldier population, who are predominantly males. As such, outcomes from this study are applicable for injured male personnel, and a separate study might be necessary to investigate if a similar metabolite profile would be observed in female patients, correspondingly, as the female gender is postulated to be protective against traumatic injuries [48]. Rather than hormonal differences, several studies have highlighted the potential contribution of genes on the X-chromosome conferring the protective benefit against trauma, one of them being Interleukin-1 receptor-associated kinase-1 (IRAK-1). IRAK-1 plays a critical role in TLR2- and TLR4-induced activation of nuclear factor (NF)-κB, leading to the downstream activation of many proinflammatory cytokines. The IRAK-1 protein has several alleles, and one of the less common variants was associated with a higher risk of developing multiple organ failure and mortality in trauma or septic patients. [49,50,51] Sperry and his co-workers demonstrated that this less common variant of IRAK-1 affected male patients more prominently because males are homozygous for this gene, whereas the heterozygous nature of the X-chromosomes meant that females are less susceptible as the detrimental effects were determined to be dose-dependent [51]. Studies are ongoing to uncover more genes that contribute to differences in trauma outcomes underlying gender differences. The pigs were also in an overnight fasted state before the injury. While this helps in ensuring reproducibility in the model, this may not fully represent the nutritional status of polytrauma patients who are usually not in a fasted state during combat. It can also have some effects on metabolism, as reported by Witowski and co-workers [52]. Lastly, the comparison of metabolic changes was only between baseline and after the 30 min shock period. The addition of more sampling at different stages of the injury phase, as well as having “injury only” and “hemorrhagic shock only” samples will better delineate the metabolic changes. 

## 4. Materials and Methods

### 4.1. Chemicals

LC-MS grade acetonitrile, ammonium formate, and formic acid were purchased from Sigma-Aldrich (St. Louis, MO, USA). Deuterium oxide (99.9 atom %D) and the NMR internal standard, 4,4-dimethyl-4-silapentane-1-sulfonic acid (DSS), were also purchased from Sigma-Aldrich. LC-MS grade methanol was purchased from Fisher Scientific (Leicestershire, UK). Ultrapure water (18.2 Ω) was obtained from a Direct-Q3 UV water purification system by Millipore (Billerica, MA, USA).

### 4.2. Animal Preparation

The pigs were subjected to a simulation of combat injury based on the porcine complex combat injury model of Cho and co-workers [53] with some modifications. The detailed experimental procedure up to the injury phase was as described in a previous publication [19]. All veterinary care and experimental procedures were in accordance with the guidelines of the Singapore National Advisory Committee for Laboratory Animal Research and were approved by the Institutional Animal Care and Use Committee of DSO National Laboratories (Protocol No. 10/108). Briefly, male Landrace-Yorkshire crossbreed swine, each weighing around 60 kg (60.3 ± 5.0 kg), were used for the study. They were fasted overnight before the experiment but were allowed ad libitum access to water until 2 h before surgery commenced. The pigs were first sedated with an intramuscular dose of atropine (50 µg/kg, Atropinsulfate, B. Braun, Melsungen, Germany), and subsequently with a 50 µL/kg cocktail of telazol-ketamine-xylazine (Telazo, Zoetis, NJ, USA; Ketamine Injection^®^, Parnell Laboratories, Auckland, New Zealand; Ilium-Xylazine-20^®^, Troy Laboratories, Sydney, Australia). The pigs were secured on the surgical table in a supine position with the four limbs securely tied to the corners of the table and intubated after reaching a stable anesthetic plane. Anesthesia was maintained using 1–3% of isoflurane. Measurement of core temperature was done using a thermometer inserted 10 cm into the rectum. Cardiac output and pulse oximetry were monitored using non-invasive continuous electro-cardiography with an Infinity Delta multi-parameter monitoring system (Dräeger, Lübeck, Germany). The mean arterial blood pressure (MABP) was also continuously monitored throughout the experiment with a blood pressure transducer and PowerLab Data Acquisition System (AD Instruments, Bella Vista, Australia). An i-STAT Handheld Blood Analyzer (Abbott Point of Care, Princeton, NJ, USA) was used to measure pH, hematocrit, base excess, partial pressure of carbon dioxide (pCO_2_), lactate, sodium, and creatinine at the designated time points. Data are presented as mean ± standard deviation (SD). The Mann–Whitney test was performed for comparison between sham and traumatized non-parametric samples (SPSS 15.0; IBM Corporation, Armonk, NY, USA). 

#### 4.2.1. Injury Phase

The midshaft of the left femur was surgically exposed, and femur fracture was introduced using the front barrel of a powder-actuated tool. Bleeding was estimated at this point based on the weight of the surgical gauze used to control the bleeding and was taken into consideration in determining the hemorrhage volume. Blood was withdrawn at a rate of 100 mL/min from the femoral artery to attain a 60% loss of the total blood volume. MABP was maintained at 25–30 mmHg through intermittent administration of isotonic 0.9% saline solution. The core temperature was reduced to 33 °C using a disposable cooling/warming torso vest to induce hypothermia. A 30-min shock period then followed to simulate the time in the field before medical intervention. 

#### 4.2.2. Blood Sampling

Blood samples were collected before the injury phase to serve as baseline samples and after the shock period, representing the after injury time point. Corresponding blood samples were also collected from sham pigs, which underwent all the experimental procedures except the injury. 

### 4.3. Sample Preparation

#### 4.3.1. Sample Preparation for NMR Analysis

Plasma samples were prepared based on a protocol by Beckonert and co-workers [54]. Two hundred microliters of plasma samples were added to a centrifuge tube containing 400 μL of 0.9% saline solution containing 10% D_2_O and 0.75 mM DSS. DSS served as a chemical shift reference and internal standard. A volume of 550 μL of each sample was transferred to 5 mm NMR tubes for analysis.

#### 4.3.2. Sample Preparation for LC-MS Analysis

One hundred microliters of plasma was added to a centrifuge tube containing 400 μL of cold methanol. The resulting mixture was allowed to stand at −80 °C for 30 min. The mixture was then centrifuged at 27,540× *g*, with the temperature maintained at 4 °C for 10 min. The supernatant was divided into two aliquots of 200 μL each and dried using a vacuum concentrator at 30 °C for 3 h. The dried samples were kept at −80 °C until analysis. Quality control (QC) samples were prepared by pooling 50 μL aliquots from each sample. Aliquots of 100 μL were taken from the pooled QC and subjected to the same extraction protocol as the other samples. 

### 4.4. Instrumental Analysis

#### 4.4.1. NMR Analysis

Proton (^1^H) NMR analysis was done using a DRX500 with Cryoprobe NMR (Bruker Biospin, Fremont, CA, USA) operating at 500.2 MHz. The Carr-Purcell-Meiboom-Gill (CPMG) pulse sequence was used to suppress the signals coming from proteins and other macromolecules. The relaxation delay used was 5 s, the spin-echo delay was 400 μs, and the acquisition time was 3 s. The peak for water with one hydrogen atom substituted with a deuterium atom (HOD peak) was identified from a basic ^1^H NMR scan, and its position was used for the solvent peak suppression before the CPMG acquisition. Data was acquired using a spectral width of 5482.5 Hz, a time-domain size of 32 k, and 128 scans per sample at 27 °C.

#### 4.4.2. Liquid Chromatography

Chromatographic separation was done on an UltiMate 3000 HPLC system (Thermo Fisher, Waltham, MA, USA). The column compartment was kept at a temperature of 40 °C, while the autosampler was at 4 °C. HILIC separation was performed using a 2.1 mm × 150 mm, 5 μm particle size ZIC-HILIC column (Merck Millipore, Darmstadt, Germany). The mobile phase consisted of 10 mM ammonium formate with 0.1% formic acid in water (A) and acetonitrile (B). The gradient program employed was: 95% B at 0–5 mins, 95–87% B from 5 to 9 mins, 87–80% B from 9 to 19 mins, 80–20% B at 19–23 mins, 20% B at 23–25 mins, followed by equilibration at 95% B for 8 mins. The flow rate was set to 0.4 mL/min, and the injection volume was 5 µL. For reverse-phase chromatography, a 2.1 mm × 100 mm Poroshell SB C18 column (Agilent Technologies, Santa Clara, CA, USA) with a particle size of 2.7 μm was used. Mobile phase A was 0.1% formic acid in water, and mobile phase B was 0.1% formic acid in acetonitrile. The gradient run consisted of an initial hold at 5% B for 2 min, increased in % B to 50% within 3 min, 50 to 98% B in 8 min, hold at 98% B for 2 min, and equilibration at 5% B for 5 min. Ten injections of QC samples were used to equilibrate the columns with the matrix at the start of the run, and one QC sample was analyzed for every batch of 10 samples. 

#### 4.4.3. Mass Spectrometry

Data acquisition was performed in both positive and negative electrospray ionization using a Triple TOF 5600 MS system (Sciex, Framingham, MA, USA). The mass range scanned was from 50 to 1000 Da with an accumulation time of 0.1 s. The nebulizer (GS1) and heater (GS2) gases were set at 50 psi, curtain (CUR) gas at 25 psi, heater temperature (TEM) at 400 °C, and ion spray voltage at 5400 volts for positive and −4500 volts for negative mode. The declustering potential and collision energy were set at 100 and 10 volts, respectively. MS/MS data were obtained through data-dependent acquisition (DDA), where the 10 highest intensity ions per cycle were subjected to a product ion scan. Declustering potential was set at 80 volts, collision energy was at 35 volts, with a collision energy spread of 15 volts. Samples were analyzed randomly to reduce instrumental drift effects. Automated mass calibration using the Sciex APCI calibration solution was done every 3 h. 

### 4.5. Data Processing and Analysis

#### 4.5.1. Data Processing for NMR Data

The ^1^H-NMR spectra were processed and profiled using Chenomx NMR Suite 7.1 (Chenomx, Edmonton, AB, Canada). Phase and baseline correction, line broadening adjustment, and HOD peak deletion were performed using the Processor module. Calibration of each spectrum was done by dragging the calibration peak and fitting its peak area to match the DSS singlet peak spectrum line at 0.00 ppm. Data binning for the untargeted analysis was done using a bin size of 0.02 ppm; the data were normalized to DSS and exported as a text file to Excel for subsequent analysis. Profiling of metabolites identifiable from the NMR spectra was also performed using the Profiler module.

#### 4.5.2. Data Pre-Processing for LC-MS

Data acquisition files from the LC-MS runs were converted to mzXML format using MSConvert (Proteowizard, Palo Alto, CA, USA) [55]. They were then uploaded to XCMS Online for retention time alignment, peak picking, and annotation. The resulting peak list was exported to Excel for further processing. Identified isotopic signals (M + 1, M + 2, etc.) were removed to reduce redundancy. Data for each sample were normalized to a constant sum. For multivariate analysis, the data were log 2 transformed to reduce variations in high-intensity signals as compared to low-intensity ones.

#### 4.5.3. Data Analysis

Multivariate analysis was done using SIMCA P+ 13 (MKS Umetrics, Sweden). Principal component analysis (PCA) was done to find inherent groupings or trends among the samples and to detect outliers. QC samples were also subjected to PCA, and Shewhart control charts were created to ensure that there were no outlying batches. If a QC sample fell outside the 3 times the standard error of the control chart and exceed its distance to model X (DModX) critical limit, it was considered as an outlier, and the samples belonging to its batch were removed from subsequent analysis. Orthogonal projection to latent structures-discriminant analysis (OPLS-DA) was performed to identify significantly differentiating features from the NMR and LC-MS data. The variable importance (VIP) values for all variables were determined for each dataset. Those with VIP values greater than 1 and with VIP cross-validation standard error (cvSE) less than the VIP value were taken as important features. Fold change values for these variables were then determined for the sham and trauma samples. The Wilcoxon-Mann-Whitney test for significance was done for the fold change values using Metaboanalyst [56]. For NMR analysis, putative identification of metabolites was done based on the chemical shift and the multiplicity of the signal at a particular bin area, searched against the Chenomx library or from literature. For LC-MS data, only those with Benjamini-Hochberg false discovery rate (FDR) of less than 5% were taken for the identification of the corresponding metabolites. The metabolites were putatively identified based on accurate mass by searching METLIN [57], HMDB [58], LipidMaps, ChemSpider [59], and MassBank [60]. Confirmation of identification was done by matching theoretical fragmentation patterns of the identified compounds with the experimental MS/MS data using PeakView 1.1.1 (Sciex, Framingham, MA, USA) and/or by matching with the MS/MS spectra available in METLIN and MassBank. A final log 2 fold change data table of metabolites found to be significantly differentiating from the NMR and LC-MS analyses was compiled. For a metabolite found to be significantly differentiating in several datasets, the data for that metabolite having the lowest *p*-value, lowest FDR, and not eluting at the void volume (for LC-MS features), was taken as part of the log 2 fold change table for pathway analysis using Metaboanalyst (Version 4.0, Xia Lab, Montreal, QC, Canada).

## 5. Conclusions

A comprehensive profile that complements the multi-faceted physiological changes occurring in injuries, particularly in cases of trauma hemorrhagic shock, has been obtained using the complementary analytical platforms of ^1^H-NMR and multi-mode LC-MS. NMR analysis revealed changes mainly associated with the activation of the acute phase of inflammation, such as decreased levels of LDLs, VLDLs, *N*-AGs, and albumin. On the other hand, multi-mode LC-MS analyses revealed changes across several metabolic pathways. An increase in levels of cysteine, methionine, and several methylated metabolites, as well as perturbation of the arginine metabolic pathway, reflect a highly active inflammatory response. Oxidative stress was observed with the decrease in lipid levels and an increase in levels of oxypurines, hypoxanthine, and xanthosine. Elevation of purine and pyrimidine bases indicates impaired energy metabolism and depletion of ATP. Lastly, the accumulation of certain uremic metabolites and toxins indicates the possible presence of acute renal injury. These results contribute to a better understanding of the mechanisms involved in trauma hemorrhagic shock, which can aid the development of diagnostic tools and metabolite supplementation strategies for the immediate care of combat trauma patients.

## Figures and Tables

**Table 1:** Survival, physiological parameters, and blood chemistry of sham and traumatized pigs.

For NMR Profiling	Sham (*n* = 4)		Trauma (*n* = 5)	
Survival, hours	48.0 ± 0.0		19.1 ± 5.6 *	
	Baseline (Mean ± SD)		30 min after Injury Phase (Mean ± SD)	
	Sham	Trauma	Sham	Trauma
Mean arterial blood pressure, mmHg	86.4 ± 10.0	81.0 ± 13.8	86.0 ± 8.9	26.7 ± 1.6 *
Core Temperature	36.7 ± 0.3	36.8 ± 0.3	35.7 ± 0.2	32.3 ± 0.7 *
pH	7.5 ± 0.1	7.5 ± 0.1	7.5 ± 0.0	7.4 ± 0.0 *
Hematocrit, %PCV	28.3 ± 5.6	26.6 ± 3.4	29.8 ± 6.3	13.6 ± 3.0 *
Base excess, mg/dL	10.8 ± 1.9	8.8 ± 4.1	10.3 ± 5.6	−1.8 ± 4.4 *
pCO_2_, mmHg	41.2 ± 4.2	47.6 ± 9.3	43.2 ± 2.7	40.4 ± 7.2
Lactate, mmol/L	1.5 ± 0.4	1.9 ± 0.4	1.6 ± 0.3	4.4 ± 1.4 *
Sodium, mmol/L	139.5 ± 1.0	140.6 ± 1.1	139.0 ± 0.8	144.0 ± 2.6 *
Chloride, mEq/L	101.5 ± 1.0	102.6 ± 2.5	101.0 ± 1.6	109.6 ± 3.0 *
**For LC-MS Profiling**	**Sham (*n* = 4)**		**Trauma (*n* = 10)**	
Survival, hours	48 ± 0.0		17.8 ± 5.1 *	
	Baseline (Mean ± SD)		30 min after injury phase (Mean ± SD)	
	Sham	Trauma	Sham	Trauma
Mean arterial blood pressure, mmHg	86.4 ± 10.0	80.1 ± 12.3	86.0 ± 8.9	26.4 ± 1.6 *
Core Temperature	36.7 ± 0.3	37.1 ± 0.4	35.7 ± 0.2	32.5 ± 1.2 *
pH	7.5 ± 0.1	7.5 ± 0.1	7.5 ± 0.1	7.3 ± 0.1 *
Hematocrit, %PCV	28.3 ± 5.6	26.7 ± 2.7	29.8 ± 6.3	15.0 ± 4.6 *
Base excess, mg/dL	10.8 ± 1.9	10.1 ± 3.2	10.3 ± 5.6	−0.8 ± 3.8 *
pCO_2_, mmHg	41.2 ± 4.2	41.5 ± 9.2	43.2 ± 2.7	35.9 ± 7.7
Lactate, mmol/L	1.5 ± 0.4	1.7 ± 0.5	1.6 ± 0.3	3.3 ± 1.6 *
Sodium, mmol/L	139.5 ± 1.0	139.8 ± 1.9	139 ± 0.8	143.3 ± 2.1 *
Chloride, mEq/L	101.5 ± 1.0	102.7 ± 2.8	101 ± 1.6	109.7 ± 2.5 *

* Significantly different from sham group (*p* < 0.05). Abbreviations: partial pressure of carbon dioxide (pCO_2_), packed cell volume (PCV).

**Table 2:** Representative differentiating metabolites identified from LC-MS analysis.

Metabolites	*m/z*/RT	Mode Detected	Other Modes ^a^	Fold Change Sham Mean (SD)	Fold Change Trauma ^b^ Mean (SD)
Glycerophospholipids and related metabolites					
Choline	104.108/13.5	H(+)		0.77 (0.17)	1.41 (0.39) **
SN-Glycero-3-phosphocholine	258.1111/23.56	H(+)		0.93 (0.19)	0.40 (0.14) **
LPC(22:5)	570.3558/10.2	R(+)	R(−)	1.09 (0.15)	0.44 (0.14) **
PA(18:4)	431.096/13.22	H(+)		0.67 (0.33)	2.51 (2.02) **
PC(16:0)	496.3407/15.25	H(+)	R(+)	0.96 (0.08)	0.58 (0.08) **
PC(22:6)	568.3386/14.95	H(+)	R(+), R(−)	1.06 (0.05)	0.46 (0.13) **
PE(20:4)	502.2919/12.37	H(+)	R(−)	1.38 (0.16)	0.67 (0.11) **
PG(20:2)	537.1672/11.2	H(+)		1.46 (0.91)	4.73 (3.93) *
PI(20:4)	619.2847/12.81	H(−)		0.87 (0.20)	0.38 (0.16) **
PS(19:0)	538.3138/9.53	R(−)		1.01 (0.04)	0.63 (0.19) **
Sphingolipids					
Cer(d18:0/16:0)	540.3653/15.64	H(+)		1.28 (0.41)	0.56 (0.17) **
C16 Sphingosine-1-phosphate	350.1488/12.72	H(−)		0.66 (0.16)	0.31 (0.11) **
Psychosine Sulfate	540.33/10.42	R(−)		1.06 (0.21)	0.55 (0.16) **
SM(d16:1/18:1)	701.5579/14.48	H(+)		1.37 (0.84)	0.66 (0.23) *
Fatty acids					
Adrenic acid	331.2628/14.04	R(−)		1.24 (0.33)	0.60 (0.23) **
Eicosadienoic acid	307.2626/14.44	R(−)		1.21 (0.56)	0.45 (0.25) **
Acylcarnitines and acylglycines					
Glutarylcarnitine	276.144/16.1	H(+)		0.87 (0.80)	6.60 (4.27) **
*N*-butyrylglycine	144.0663/2.91	H(−)		0.69 (0.22)	2.48 (3.09) **
Phenylacetylgycine ^d^	192.0668/5.47	R(−)	H(+)	0.83 (0.15)	2.09 (0.64) **
Hippuric Acid (Benzoylglycine) ^c^	178.0513/4.29	R(−)	R(+), H(+)	0.75 (0.11)	1.78 (0.44) **
Amino acids and derivatives					
Citrulline	176.1029/23.1	H(+)		1.07 (0.17)	1.40 (0.30) *
l-Methionine	133.0316/14.92	H(+)		0.94 (0.06)	1.34 (0.39) **
*S*-Adenosylmethionine	399.1448/24.54	H(+)		0.98 (0.18)	1.99 (0.71) **
Cysteine-Homocysteine disulfide	253.0315/23.94	H(−)		0.95 (0.18)	2.55 (0.93) **
l-Cysteinylglycine disulfide	298.053/24.42	H(+)		0.93 (0.19)	1.98 (0.95) **
l-Cystine	241.0314/24.1	H(+)	R(+)	0.95 (0.11)	1.60 (0.70) **
N1,N12-Diacetylspermine	287.2446/25.22	H(+)		1.10 (0.35)	2.22 (0.86) **
N1-acetylspermidine	188.1763/25.51	H(+)		1.09 (0.27)	2.34 (1.23) **
Pantothenic Acid	218.103/1.64	R(−)		0.87 (0.07)	1.37 (0.27) **
Phenylethylamine ^c^	105.034/2.73	H(+)		0.72 (0.20)	1.35 (0.42) **
2,4-Diamino-butyric acid	117.0199/1.01	R(−)		0.79 (0.20)	2.70 (1.65) **
2,6-Diamino-heptanedioic acid	189.0404/0.96	R(−)		0.93 (0.17)	1.67 (0.71) **
4-(2-Aminophenyl)-2,4-dioxobutanoic acid	206.0457/4.62	R(−)	R(+), H(+)	1.03 (0.25)	2.13 (0.71) **
Methylated metabolites					
1-Methylguanosine ^c^	298.114/1.24	R(+)	H(+)	1.13 (0.09)	1.54 (0.38) **
1-methyluric Acid	181.0359/10.41	H(−)		0.99 (0.33)	2.25 (0.67) **
3’-*O*-Methyladenosine	282.1196/17.53	H(+)		0.99 (0.23)	1.46 (0.37) **
Purines					
Adenosine	250.0928/1.94	H(+)		0.87 (0.32)	2.71 (2.10) *
Deoxyguanosine	266.0878/12.32	H(−)		1.43 (0.67)	6.43 (3.29) **
Guanosine	282.0829/14.25	H(−)	R(+), R(−)	1.22 (0.50)	3.73 (2.09) **
Hypoxanthine ^c^	137.0462/11.17	H(−)	H(+)	1.00 (0.28)	3.08 (1.99) **
Inosine ^c^	269.0876/11.18	H(+)	R(+), R(−)	1.38 (0.62)	3.77 (2.24) **
Succinoadenosine	382.0993/1.79	R(−)		1.03 (0.15)	2.09 (0.61) **
Uric Acid ^c^	169.0348/14.69	H(+)	R(−)	0.72 (0.32)	5.43 (4.78) **
Xanthosine ^c^	285.0818/12.17	H(+)	R(−)	1.00 (0.35)	2.65 (0.95) **
Pyrimidines					
Cytosine	112.0505/9.76	H(+)		0.61 (0.16)	1.66 (0.52) **
Cytidine ^c^	226.0817/9.78	H(+)		0.94 (0.14)	1.72 (0.49) **
Cytidine Monophosphate	322.2006/1.33	H(−)		0.97 (0.16)	0.54 (0.16) **
Thymidine	241.0824/1.38	R(−)		1.00 (0.17)	1.82 (0.43) **
Thymine ^c^	127.05/1.77	H(+)		1.10 (0.21)	1.64 (0.34) *
Uridine	243.0618/0.92	R(−)		0.81 (0.19)	2.07 (0.73) **
Glucuronides					
3-indolecarboxylic acid glucuronide	338.087/19	H(+)		0.80 (0.37)	2.33 (1.21) **
*p*-Cresol glucuronide ^d^	283.0821/5.93	R(−)	H(−), R(+)	0.86 (0.23)	2.22 (0.71) **
Phenethylamine glucuronide ^d^	342.1186/1.07	R(−)		1.16 (0.14)	3.59 (1.93) *

^a^ Other LC-MS modes, the same metabolite feature was detected. Abbreviations: mass to charge ratio/retention time (mins)—*m*/*z*/RT; H—HILIC LC-MS; R—Reverse Phase LC-MS; (+)—Positive Ionization Mode; (−)—Negative Ionization Mode; ^b^ Significant based on Wilcoxon–Mann–Whitney significance test of fold change values for Sham vs. Trauma: * *p* < 0.05 ** *p* < 0.01. ^c^ Identified as uremic retention solutes from the European Uremia Toxin (EuTox) Work Group database. ^d^ Parent metabolite (metabolite before glucuronidation) identified as uremic toxins from the EuTox Work Group database.

## Supplementary Materials

The following are available online at https://www.mdpi.com/2218-1989/10/9/373/s1, Table S1: Fold-change comparison of significant features from NMR binned data (*p* < 0.05), Table S2: Comparison of profiled metabolite concentrations using Chenomx Profiler, Table S3: Fold-change values of LC-MS metabolites identified to be significantly changed after trauma, Table S4: Top metabolic pathways affected after the trauma injury, Figure S1: Comparison of the base peak chromatograms (BPCs) of the HILIC-LC-MS analyses for the sham and trauma samples, Figure S2: Comparison of the BPCs of the RP-LC-MS analyses for the sham and trauma samples, Figure S3: Shewhart control charts of the LC-MS pooled QC samples.

## References

[1] Lozano R., Naghavi M., Foreman K., Lim S., Shibuya K., Aboyans V., Abraham J., Adair T., Aggarwal R., Ahn S.Y. (2012). Global and regional mortality from 235 causes of death for 20 age groups in 1990 and 2010: A systematic analysis for the Global Burden of Disease Study 2010. Lancet.

[2] Belmont P.J., Goodman G.P., Zacchilli M., Posner M., Evans C., Owens B.D. (2010). Incidence and Epidemiology of Combat Injuries Sustained During “The Surge” Portion of Operation Iraqi Freedom by a U.S. Army Brigade Combat Team. J. Trauma Inj. Infect. Crit. Care.

[3] Vuoncino M., Hoo A.J.S., Patel J.A., White P.W., Rasmussen T.E., White J.M. (2020). Epidemiology of Upper Extremity Vascular Injury in Contemporary Combat. Ann. Vasc. Surg..

[4] Angele M.K., Schneider C.P., Chaudry I.H. (2008). Bench-to-bedside review: Latest results in hemorrhagic shock. Crit. Care.

[5] Fröhlich M., Lefering R., Probst C., Paffrath T., Schneider M.M., Maegele M., Sakka S.G., Bouillon B., Wafaisade A. (2014). Epidemiology and risk factors of multiple-organ failure after multiple trauma. J. Trauma Acute Care Surg..

[6] Sauaia A., Moore E.E., Johnson J.L., Chin T.L., Banerjee A., Sperry J.L., Maier R.V., Burlew C.C. (2014). Temporal trends of postinjury multiple-organ failure. J. Trauma Acute Care Surg..

[7] El Sayad M., Noureddine H. (2014). Recent Advances of Hemorrhage Management in Severe Trauma. Emerg. Med. Int..

[8] Mabry R., McManus J.G. (2008). Prehospital advances in the management of severe penetrating trauma. Crit. Care Med..

[9] Fiehn O. (2002). Metabolomics—The link between genotypes and phenotypes. Plant Mol. Biol..

[10] Serkova N.J., Standiford T.J., Stringer K.A. (2011). The Emerging Field of Quantitative Blood Metabolomics for Biomarker Discovery in Critical Illnesses. Am. J. Respir. Crit. Care Med..

[11] Lexcen D.R., Lusczek E.R., Witowski N.E., Mulier K.E., Beilman G.J. (2012). Metabolomics classifies phase of care and identifies risk for mortality in a porcine model of multiple injuries and hemorrhagic shock. J. Trauma Acute Care Surg..

[12] Lusczek E.R., Lexcen D.R., Witowski N.E., Mulier K.E., Beilman G., Beilman G.J. (2012). Urinary metabolic network analysis in trauma, hemorrhagic shock, and resuscitation. Metabolomics.

[13] Lusczek E.R., Lexcen D.R., Witowski N.E., Determan C., Mulier K.E., Beilman G.J. (2014). Prolonged Induced Hypothermia in Hemorrhagic Shock Is Associated with Decreased Muscle Metabolism. Shock.

[14] Bogren L.K., Murphy C.J., Johnston E.L., Sinha N., Serkova N.J., Drew K.L. (2014). 1H–NMR Metabolomic Biomarkers of Poor Outcome after Hemorrhagic Shock are Absent in Hibernators. PLoS ONE.

[15] Lusczek E.R., Muratore S.L., Dubick M.A., Beilman G.J. (2017). Assessment of key plasma metabolites in combat casualties. J. Trauma Acute Care Surg..

[16] D’alessandro A., Moore H.B., Moore E.E., Wither M.J., Nemkov T., Gonzalez E., Slaughter A., Fragoso M., Hansen K.C., Silliman C.C. (2015). Early hemorrhage triggers metabolic responses that build up during prolonged shock. Am. J. Physiol. Integr. Comp. Physiol..

[17] Chouchani E.T., Pell V.R., Gaude E., Aksentijević D., Sundier S.Y., Robb E.L., Logan A., Nadtochiy S.M., Ord E.N., Smith A.C. (2014). Ischaemic accumulation of succinate controls reperfusion injury through mitochondrial ROS. Nature.

[18] Slaughter A.L., Nunns G.R., D’alessandro A., Banerjee A., Hansen K.C., Moore E.E., Silliman C.C., Nemkov T., Moore H.B., Fragoso M. (2018). The Metabolopathy of Tissue Injury, Hemorrhagic Shock, and Resuscitation in a Rat Model. Shock.

[19] Wong Y.C., Lai Y.Y., Tan M.H., Tan C.S., Wu J., Zeng L.Z.J., Lu J., Moochhala S.M. (2015). Potential Biomarker Panel for Predicting Organ Dysfunction and Acute Coagulopathy in a Polytrauma Porcine Model. Shock.

[20] Nicholson J.K., Foxall P.J.D., Spraul M., Farrant R.D., Lindon J.C. (1995). 750 MHz 1H and 1H-13C NMR Spectroscopy of Human Blood Plasma. Anal. Chem..

[21] McGill M.R., Li F., Sharpe M.R., Williams C.D., Curry S.C., Ma X., Jaeschke H. (2013). Circulating acylcarnitines as biomarkers of mitochondrial dysfunction after acetaminophen overdose in mice and humans. Arch. Toxicol..

[22] Newgard C.B. (2012). Interplay between lipids and branched-chain amino acids in development of insulin resistance. Cell Metab..

[23] Tsukamoto T., Pape H.C. (2009). Animal models for trauma research. Shock.

[24] Merrifield C.A., Lewis M.C., Claus S.P., Beckonert O.P., Dumas M.-E., Duncker S., Kochhar S., Rezzi S., Lindon J.C., Bailey M. (2011). A metabolic system-wide characterisation of the pig: A model for human physiology. Mol. BioSyst..

[25] Tremoleda J.L., Watts S.A., Reynolds P.S., Thiemermann C., Brohi K. (2017). Modeling Acute Traumatic Hemorrhagic Shock Injury. Shock.

[26] Reisz J.A., Wither M.J., Moore E.E., Slaughter A.L., Moore H.B., Ghasabyan A., Chandler J., Schaub L.J., Fragoso M., Nunns G. (2018). All animals are equal but some animals are more equal than others. J. Trauma Acute Care Surg..

[27] Scribner D.M., Witowski N.E., Mulier K.E., Lusczek E.R., Wasiluk K.R., Beilman G.J. (2010). Liver Metabolomic Changes Identify Biochemical Pathways in Hemorrhagic Shock. J. Surg. Res..

[28] Simsek T., Simsek H.U., Cantürk N.Z. (2014). Response to trauma and metabolic changes: Posttraumatic metabolism. Turk. J. Surg..

[29] Kumaraswamy S.B., Linder A., Åkesson P., Dahlbäck B. (2012). Decreased plasma concentrations of apolipoprotein M in sepsis and systemic inflammatory response syndromes. Crit. Care.

[30] Manani S.M., Virzì G.M., Clementi A., Brocca A., De Cal M., Tantillo I., Ferrando L., Crepaldi C., Ronco C. (2015). Pro-inflammatory cytokines: A possible relationship with dialytic adequacy and serum albumin in peritoneal dialysis patients. Clin. Kidney J..

[31] Wang Y., Holmes E., Tang H., Lindon J.C., Sprenger N., Turini M.E., Bergonzelli G., Fay L.B., Kochhar S., Nicholson J.K. (2006). Experimental Metabonomic Model of Dietary Variation and Stress Interactions. J. Proteome Res..

[32] Preiser J.-C., Ichai C., Orban J.-C., Groeneveld A.B.J. (2014). Metabolic response to the stress of critical illness. Br. J. Anaesth..

[33] Liochev S.I. (2013). Reactive oxygen species and the free radical theory of aging. Free. Radic. Boil. Med..

[34] Yang H., Wang H., Chavan S.S., Andersson U. (2015). High Mobility Group Box Protein 1 (HMGB1): The Prototypical Endogenous Danger Molecule. Mol. Med..

[35] Lee J.S., Wang R.X., Alexeev E.E., Lanis J.M., Battista K.D., Glover L., Colgan S.P. (2018). Hypoxanthine is a checkpoint stress metabolite in colonic epithelial energy modulation and barrier function. J. Boil. Chem..

[36] Bin P., Huang R., Zhou X. (2017). Oxidation Resistance of the Sulfur Amino Acids: Methionine and Cysteine. BioMed Res. Int..

[37] Pfalzer A.C., Choi S.-W., Tammen S.A., Park L.K., Bottiglieri T., Parnell L.D., Lamon-Fava S. (2014). S-adenosylmethionine mediates inhibition of inflammatory response and changes in DNA methylation in human macrophages. Physiol. Genom..

[38] Wong J., Piceno Y.M., DeSantis T.Z., Pahl M., Andersen G.L., Vaziri N.D. (2014). Expansion of urease- and uricase-containing, indole- and p-cresol-forming and contraction of short-chain fatty acid-producing intestinal microbiota in ESRD. Am. J. Nephrol..

[39] Yamamoto S., Fukagawa M. (2017). Uremic Toxicity and Bone in CKD. J. Nephrol..

[40] Lisowska-Myjak B. (2014). Uremic Toxins and Their Effects on Multiple Organ Systems. Nephron Clin. Pract..

[41] Saito H., Yoshimura M., Saigo C., Komori M., Nomura Y., Yamamoto Y., Sagata M., Wakida A., Chuman E., Nishi K. (2014). Hepatic Sulfotransferase as a Nephropreventing Target by Suppression of the Uremic Toxin Indoxyl Sulfate Accumulation in Ischemic Acute Kidney Injury. Toxicol. Sci..

[42] Zarbock A., Gomez H., A Kellum J. (2014). Sepsis-induced acute kidney injury revisited. Curr. Opin. Crit. Care.

[43] Liang X., Chen Y., Zhang L., Jiang F., Wang W., Ye Z., Liu S., Yu C., Shi W. (2014). Necroptosis, a novel form of caspase-independent cell death, contributes to renal epithelial cell damage in an ATP-depleted renal ischemia model. Mol. Med. Rep..

[44] Jackson E.K., Cheng N., Mi Z., Gillespie D.G. (2014). Guanosine regulates adenosine levels in the kidney. Physiol. Rep..

[45] D’alessandro A., Moore H.B., Moore E.E., Reisz J.A., Wither M.J., Ghasasbyan A., Chandler J., Silliman C.C., Hansen K.C., Banerjee A. (2017). Plasma succinate is a predictor of mortality in critically injured patients. J. Trauma Acute Care Surg..

[46] D’alessandro A., Slaughter A.L., Peltz E.D., Moore E.E., Silliman C.C., Wither M.J., Nemkov T., Bacon A.W., Fragoso M., Banerjee A. (2015). Trauma/hemorrhagic shock instigates aberrant metabolic flux through glycolytic pathways, as revealed by preliminary (13)C-glucose labeling metabolomics. J. Transl. Med..

[47] Clendenen N., Nunns G.R., Moore E.E., Reisz J.A., Gonzalez E., Peltz E., Silliman C.C., Fragoso M., Nemkov T., Wither M.J. (2017). Hemorrhagic shock and tissue injury drive distinct plasma metabolome derangements in swine. J. Trauma Acute Care Surg..

[48] Sethuraman K.N., Marcolini E.G., McCunn M., Hansoti B., Vaca F.E., Napolitano L.M. (2014). Gender-specific issues in traumatic injury and resuscitation: Consensus-based recommendations for future research. Acad. Emerg. Med..

[49] Toubiana J., Courtine E., Pene F., Viallon V., Asfar P., Daubin C., Rousseau C., Chenot C., Ouaaz F., Grimaldi D. (2010). IRAK1 functional genetic variant affects severity of septic shock. Crit. Care Med..

[50] Arcaroli J., Silva E., Maloney J.P., He Q., Svetkauskaite D., Murphy J.R., Abraham E. (2006). Variant IRAK-1 Haplotype Is Associated with Increased Nuclear Factor–κB Activation and Worse Outcomes in Sepsis. Am. J. Respir. Crit. Care Med..

[51] Sperry J.L., Zolin S., Zuckerbraun B.S., Vodovotz Y., Namas R.A., Neal M.D., Ferrell R.E., Rosengart M.R., Peitzman A.B., Billiar T.R. (2014). X Chromosome-Linked IRAK-1 Polymorphism Is a Strong Predictor of Multiple Organ Failure and Mortality Postinjury. Ann. Surg..

[52] Witowski N., Lusczek E.R., Determan C., Lexcen D., Mulier K., Ostrowski B., Beilman G.J. (2015). A Four-Compartment Metabolomics Analysis of the Liver, Muscle, Serum, and Urine Response to Polytrauma with Hemorrhagic Shock following Carbohydrate Prefeed. PLoS ONE.

[53] Cho S.D., Holcomb J.B., Tieu B.H., Englehart M.S., Morris M.S., Karahan Z.A., Underwood S.A., Muller P.J., Prince M.D., Medina L. (2009). Reproducibility of an animal model simulating complex combat-related injury in a multiple-institution format. Shock.

[54] Beckonert O., Keun H.C., Ebbels T.M.D., Bundy J.G., Holmes E., Lindon J.C., Nicholson J.K. (2007). Metabolic profiling, metabolomic and metabonomic procedures for NMR spectroscopy of urine, plasma, serum and tissue extracts. Nat. Protoc..

[55] Chambers M.C., MacLean B., Burke R., Amodei D., Ruderman D.L., Neumann S., Gatto L., Fischer B., Pratt B., Egertson J. (2012). A cross-platform toolkit for mass spectrometry and proteomics. Nat. Biotechnol..

[56] Xia J., Mandal R., Sinelnikov I.V., Broadhurst D.I., Wishart D.S. (2012). MetaboAnalyst 2.0--a comprehensive server for metabolomic data analysis. Nucleic Acids Res..

[57] Tautenhahn R., Cho K., Uritboonthai W., Zhu Z., Patti G.J., Siuzdak G. (2012). An accelerated workflow for untargeted metabolomics using the METLIN database. Nat. Biotechnol..

[58] Wishart D.S., Jewison T., Guo A.C., Wilson M., Knox C., Liu Y., Djoumbou Y., Mandal R., Aziat F., Dong E. (2012). HMDB 3.0—The Human Metabolome Database in 2013. Nucleic Acids Res..

[59] Pence H.E., Williams A.J. (2010). ChemSpider: An Online Chemical Information Resource. J. Chem. Educ..

[60] Horai H., Arita M., Kanaya S., Nihei Y., Ikeda T., Suwa K., Ojima Y., Tanaka K., Tanaka S., Aoshima K. (2010). MassBank: A public repository for sharing mass spectral data for life sciences. J. Mass Spectrom..

