# Supplementary material for: Metabolic Profiling of a Porcine Combat Trauma-Injury Model Using NMR and Multi-Mode LC-MS Metabolomics—A Preliminary Study
Source: Metabolites. 2020 Sep 16;10(9):373. doi: 10.3390/metabo10090373 (PMC7570375; doi:10.3390/metabo10090373)
Supplement: Supplementary file 1 [file metabolites-10-00373-s001.pdf]

Supporting Information

Metabolic Profiling of a Porcine Combat Trauma-Injury Model Using NMR and Multi-Mode LC-MS Metabolomics—A Preliminary Study

Anna Karen Carrasco Laserna [1], Yiyang Lai [2], Guihua Fang [1,3], Rajaseger Ganapathy [2], Mohamed Shirhan Bin Mohamed Atan [4], Jia Lu [2], Jian Wu [2], Mahesh Uttamchandani [1,2], Shabbir M Moochhala [4,5,*] and Sam Fong Yau Li [1,6,*]

1. Department of Chemistry, Faculty of Science, National University of Singapore, 3 Science Drive 3, Singapore 117543, Singapore, chmakcl@nus.edu.sg (A.K.C.L.)
2. Defence Medical and Environmental Research Institute, DSO National Laboratories, 27 Medical Drive, Singapore 117510, Singapore, lyiyang@dso.org.sg (Y.L.); grajaseg@dso.org.sg (R.G.); ljia@dso.org.sg (J.L.); wjian@dso.org.sg (J.W.); umahesh@dso.org.sg (M.U.)
3. Forensic Science Division, Health Services Authority, 11 Outram Road, Singapore 169078, Singapore, fang_guihua@hsa.gov.sg (G.F.)
4. School of Applied Sciences, Temasek Polytechnic, 21 Tampines Ave 1, Singapore 529757, Singapore, shirhana@tp.edu.sg (M.S.B.M.A.)
5. Department of Pharmacology, Yong Loo Lin School of Medicine, National University of Singapore, Blk MD3, 16 Medical Drive, Singapore 117600, Singapore
6. NUS Environmental Research Institute, National University of Singapore, T-Lab Building, 5A Engineering Drive 1, Singapore 117411, Singapore

* Correspondence: phcsmm@nus.edu.sg (S.M.M.), chmlifys@nus.edu.sg (S.F.Y.L.); Tel.: +65-6516-2681 (S.F.Y.L.)

Received: 30 May 2020; Accepted: 10 September 2020; Published: date

Table S1. Fold-change comparison of significant features from NMR binned data ($p < 0.05$).

Chemical shift (ppm)	Multiplicity	Molecule	Assignment	FC SHAM Mean (SD)	FC TRAUMA Mean (SD)
0.75	m	Cholesterol	C26 and C27	1.01 (0.22)	0.51 (0.1)
0.77	m	Cholesterol	C26 and C27	1.01 (0.19)	0.49 (0.07)
0.79	m	Cholesterol	C26 and C27	1.01 (0.19)	0.48 (0.05)
0.81	m	Lipids (Mainly LDL)	$CH_3(CH_2)_n$	1.04 (0.25)	0.49 (0.06)
0.83	m	Lipids (Mainly LDL)	$CH_3(CH_2)_n$	1.01 (0.19)	0.47 (0.06)
0.85	m	Lipids (Mainly VLDL)	$CH_3CH_2CH_2C=$	0.95 (0.15)	0.46 (0.06)
0.87	m	Lipids (Mainly VLDL)	$CH_3CH_2CH_2C=$	0.96 (0.16)	0.49 (0.06)
0.89	m	Lipids (Mainly VLDL)	$CH_3CH_2CH_2C=$	0.98 (0.14)	0.48 (0.06)
0.91		Cholesterol	C21	0.96 (0.17)	0.53 (0.09)
0.93	m	Lipids	δ-CH_3/CH_3CH_2	1.02 (0.19)	0.59 (0.09)
0.95	d	Leucine/Lipids	δ-CH_3/CH_3CH_2	1.02 (0.17)	0.76 (0.14)
1.19	m	Lipids	$CH_3CH_2CH_2$	0.91 (0.18)	0.47 (0.04)
1.21	m	Lipids	$CH_3CH_2CH_2$	1 (0.18)	0.49 (0.08)
1.23	m	Lipids	$CH_3CH_2CH_2$	0.96 (0.14)	0.48 (0.07)
1.25	m	Lipids (Mainly LDL)	$(CH_2)_n$	0.96 (0.14)	0.51 (0.06)
1.27	m	Lipids	$CH_3CH_2(CH_2)_n$	1.05 (0.17)	0.52 (0.05)
1.29	m	Lipids (Mainly VLDL)	$CH_2CH_2CH_2CO$	0.98 (0.15)	0.51 (0.06)
1.39		Unknown		0.9 (0.18)	0.45 (0.11)
1.41		Unknown		0.94 (0.15)	0.44 (0.1)
1.43		Unknown		0.91 (0.15)	0.53 (0.17)

1.57	m	Lipids (mainly VLDL)	CH_2CH_2CO	1.09 (0.3)	0.47 (0.08)
1.59	m	Lipids/Citrulline	CH_2CH_2CO/γ-CH_2	1.03 (0.22)	0.43 (0.08)
1.61	m	Lipids/Citrulline	CH_2CH_2CO/γ-CH_2	1.01 (0.21)	0.41 (0.09)
1.65	m	Arginine	γ-CH_2	1.04 (0.25)	0.51 (0.07)
1.67	m	Arginine/Lipids	γ-$CH_2/CH_2CH_2C=C$	1.02 (0.19)	0.54 (0.07)
1.69	m	Lysine/Lipids	δ-CH_2/ $CH_2CH_2C=C$	1.02 (0.19)	0.63 (0.11)
1.71	m	Leucine/Lipids	β-CH_2, γ-CH/ $CH_2CH_2C=C$	1.03 (0.18)	0.75 (0.12)
1.73	m	Leucine/Lipids	β-CH_2, γ-CH/ $CH_2CH_2C=C$	1.01 (0.13)	0.76 (0.12)
1.93	s	Acetate	CH_3	0.9 (0.12)	0.5 (0.17)
1.95	m	Lipids	$CH_2C=C$	1.08 (0.26)	0.47 (0.07)
1.97	m	Lipids	$CH_2C=C$	1.04 (0.23)	0.52 (0.08)
1.99	m	Lipids	$CH_2C=C$	1.03 (0.19)	0.54 (0.07)
2.01	m	Lipids	$CH_2C=C$	1.01 (0.17)	0.53 (0.06)
2.03	s	Glycoproteins (N-acetyl)	$NHCOCH_3$	1.08 (0.3)	0.51 (0.07)
2.05	m	Proline	half β-CH_2	0.92 (0.15)	0.43 (0.08)
2.07	s	Glycoproteins (N-acetyl)	$OCOCH_3$	0.99 (0.18)	0.49 (0.08)
2.09	m	Glutamine	half γ-CH_2	0.97 (0.27)	0.52 (0.11)
2.25	m	Valine/Lipids	β-CH/ CH_2CO	1.05 (0.23)	0.54 (0.1)
2.27	m	Valine/Lipids	β-CH/ CH_2CO	1 (0.18)	0.56 (0.11)
2.29	m	Valine/Lipids	β-CH/ CH_2CO	0.97 (0.1)	0.5 (0.14)
2.31	m	Valine/Lipids	β-CH/ CH_2CO	0.97 (0.11)	0.48 (0.13)
2.33	m	Glutamate	half γ-CH_2	1.01 (0.2)	0.68 (0.13)
2.67	d/m	Citrate/Lipids	half CH_2/ $C=CCH_2C=C$	1.04 (0.15)	0.54 (0.18)
2.69	d/m	Citrate/Lipids	half CH_2/ $C=CCH_2C=C$	1.01 (0.16)	0.56 (0.13)
2.71	d/m	Citrate/Lipids	half CH_2/ $C=CCH_2C=C$	0.94 (0.19)	0.49 (0.15)
2.73	m	Lipids	half CH_2/ $C=CCH_2C=C$	1.06 (0.3)	0.5 (0.11)
2.75	m	Lipids	half CH_2/ $C=CCH_2C=C$	1.03 (0.28)	0.48 (0.11)
2.93	t	Albumin (lysyl)	ϵ-CH_2	0.97 (0.26)	0.59 (0.13)
2.95	t	Albumin (lysyl)	ϵ-CH_2	0.99 (0.23)	0.51 (0.11)
2.97	t	Albumin (lysyl)	ϵ-CH_2	0.99 (0.21)	0.49 (0.11)
2.99	t	Albumin (lysyl)	ϵ-CH_2	0.97 (0.19)	0.48 (0.1)
3.01	t	Albumin (lysyl)	ϵ-CH_2	0.98 (0.19)	0.55 (0.12)
3.07	dd	Tyrosine	half β-CH_2	0.96 (0.25)	0.5 (0.11)
3.11	dd	Phenylalanine	half β-CH_2	1.04 (0.3)	0.6 (0.19)
3.21	s	Choline	$N(CH_3)_3$	1.05 (0.24)	0.55 (0.07)
3.27	s/dd	Betaine/Glucose	$N(CH_3)_3/H_2$ of β-Glucose	0.98 (0.14)	0.52 (0.19)
3.55	s/dd	Glycine/Glucose	CH_2/ H_2 of α-Glucose	1.00 (0.17)	0.75 (0.11)
3.57	d/m	Valine/unknown	α-CH	0.9 (0.13)	0.5 (0.08)
3.61	d/m	Threonine/ unknown	α-CH	1.01 (0.15)	0.62 (0.11)
3.63		Unknown		1.04 (0.25)	0.56 (0.05)
3.65	m	SN-GPC	NCH_2	0.99 (0.28)	0.55 (0.05)
3.67	m	SN-GPC	NCH_2	0.94 (0.18)	0.51 (0.06)
3.79	dd	Glucose	Half CH_2-C6 of α-Glucose	1.04 (0.31)	0.69 (0.14)
3.87	m	Glucose	Half CH_2-C6 of α-Glucose	1.01 (0.18)	0.6 (0.1)
3.95	dd/s	Glucose/Creatine	Half CH_2-C6 of β-glucose	0.96 (0.23)	0.6 (0.1)
3.97	m	Phenylalanine	α-CH	0.99 (0.03)	0.59 (0.09)
3.99	m	Phenylalanine/ Lipids	α-CH/ CH_2OCOR	0.98 (0.08)	0.55 (0.07)
4.29	m	Lipids	OCH_2	1.06 (0.27)	0.51 (0.09)
4.31	m	Lipids	OCH_2	0.93 (0.17)	0.48 (0.09)
5.25	m	Unsaturated Lipids	$CH=CHCH_2CH=CH$	0.99 (0.21)	0.51 (0.05)
5.27	m	Unsaturated Lipids	$=CHCH_2CH_2$	1.02 (0.17)	0.5 (0.11)
5.29	m	Unsaturated Lipids	$CH=CHCH_2CH=CH$	1.01 (0.16)	0.48 (0.1)
5.31	m	Unsaturated Lipids	$=CHCH_2CH_2$	1 (0.19)	0.49 (0.14)

Table S2. Comparison of profiled metabolite concentrations using Chenomx Profiler.

Metabolite	Concentration (mM)					
	Sham			Trauma		
	Before Mean (SD)	After Mean (SD)	FC Mean (SD)	Before Mean (SD)	After Mean (SD)	FC Mean (SD)
3-Hydroxybutyrate	0.067 (0.038)	0.034 (0.013)	0.62 (0.342)	0.028 (0.018)	0.034 (0.011)	9.596 (19.16)
3-Hydroxyisovalerate	0.013 (0.001)	0.009 (0.005)	0.676 (0.300)	0.013 (0.002)	0.007 (0.005)	0.506 (0.313)
Acetate	0.125 (0.062)	0.108 (0.056)	0.883 (0.205)	0.175 (0.058)	0.186 (0.034)	1.146 (0.38)
Acetone	0.069 (0.066)	0.061 (0.054)	1.106 (0.362)	0.07 (0.051)	0.046 (0.037)	0.618 (0.215)
Alanine	0.424 (0.048)	0.453 (0.098)	1.069 (0.221)	0.461 (0.094)	0.439 (0.073)	0.989 (0.283)
Arginine	0.261 (0.038)	0.253 (0.052)	0.988 (0.299)	0.239 (0.037)	0.154 (0.02)	0.661 (0.153)*
Betaine	0.112 (0.028)	0.096 (0.042)	0.827 (0.194)	0.13 (0.073)	0.08 (0.04)	0.61 (0.181)
Choline	0 (0)	0.001 (0.002)	2.25 (2.50)	0 (0)	0.009 (0.002)	12.213 (2.562)*
Citrate	0.14 (0.029)	0.13 (0.022)	0.938 (0.076)	0.166 (0.022)	0.126 (0.033)	0.754 (0.131)
Creatine	0.266 (0.149)	0.257 (0.137)	1.078 (0.447)	0.168 (0.079)	0.136 (0.091)	0.75 (0.248)
Creatinine	0.136 (0.028)	0.125 (0.041)	0.904 (0.147)	0.106 (0.008)	0.101 (0.018)	0.96 (0.181)
Formate	0.009 (0.011)	0.016 (0.005)	9.75 (10.252)	0.005 (0.007)	0.014 (0.007)	10.286 (11.892)
Glucose	2.963 (0.622)	2.781 (0.698)	0.981 (0.34)	3.9 (0.989)	3.886 (0.93)	1.036 (0.332)
Glutamate	0.2 (0.038)	0.169 (0.053)	0.853 (0.257)	0.184 (0.047)	0.162 (0.032)	0.899 (0.151)
Glutamine	0.33 (0.026)	0.297 (0.012)	0.907 (0.107)	0.38 (0.042)	0.285 (0.046)	0.756 (0.155)
Glycine	1.12 (0.227)	1.041 (0.348)	0.919 (0.149)	1.011 (0.142)	0.747 (0.172)	0.74 (0.153)
Hypoxanthine	0.004 (0.009)	0 (0)	0.761 (0.478)	0 (0)	0.01 (0.011)	14.027 (14.352)
Isoleucine	0.127 (0.032)	0.13 (0.027)	1.059 (0.287)	0.09 (0.019)	0.09 (0.018)	1.022 (0.24)
Lactate	1.493 (0.231)	2.073 (1.418)	1.472 (1.202)	1.446 (0.397)	2.328 (1.137)	1.758 (1.022)
Leucine	0.136 (0.027)	0.15 (0.015)	1.121 (0.117)	0.141 (0.049)	0.125 (0.025)	0.958 (0.348)
Lysine	0.085 (0.029)	0.081 (0.03)	1.026 (0.358)	0.065 (0.038)	0.09 (0.029)	2.726 (3.254)
Methionine	0.046 (0.015)	0.04 (0.006)	0.913 (0.217)	0.038 (0.009)	0.032 (0.009)	0.864 (0.28)
N-Nitrosodimethyl-amine	0.034 (0.016)	0.028 (0.017)	0.79 (0.235)	0.027 (0.006)	0.019 (0.008)	0.706 (0.164)
Phenylalanine	0.057 (0.01)	0.055 (0.013)	0.979 (0.185)	0.04 (0.011)	0.058 (0.02)	1.505 (0.538)
Proline	0.226 (0.029)	0.218 (0.066)	0.996 (0.400)	0.168 (0.076)	0.139 (0.029)	1.358 (1.507)
Pyruvate	0.101 (0.013)	0.129 (0.077)	1.337 (0.983)	0.122 (0.026)	0.113 (0.026)	0.961 (0.285)
Succinate	0.004 (0.003)	0.006 (0.001)	1.932 (1.125)	0.005 (0.003)	0.017 (0.02)	2.937 (2.02)
Threonine	0.211 (0.017)	0.164 (0.029)	0.776 (0.121)	0.169 (0.059)	0.109 (0.021)	0.683 (0.18)
Tyrosine	0.063 (0.009)	0.062 (0.006)	0.991 (0.146)	0.055 (0.003)	0.056 (0.01)	1.027 (0.18)
Urea	0.831 (0.629)	0.771 (0.656)	1.149 (0.62)	0.226 (0.224)	0.215 (0.481)	0.816 (0.863)
Valine	0.267 (0.06)	0.259 (0.029)	1.007 (0.234)	0.207 (0.041)	0.179 (0.027)	0.885 (0.201)

* Significant based on Wilcoxon-Mann-Whitney significance test of fold change values ($p < 0.05$).

Table S3. Fold change values of LC-MS metabolites identified to be significantly changed after trauma.

Metabolite ID	MZ/RT	Mode	Other modes	FC Sham Mean (SD)	FC Trauma[a] Mean (SD)
Citrulline	176.1029/23.1	H(+)		1.07 (0.17)	1.4 (0.3)*
L-methionine	133.0316/14.92	H(+)		0.94 (0.06)	1.34 (0.39)**
4-Methylene-L-glutamine	159.0768/23.1	H(+)		1.06 (0.1)	1.33 (0.32)**
Cysteine-Homocysteine disulfide	253.0315/23.94	H(-)		0.95 (0.18)	2.55 (0.93)**
L-Cysteinylglycine disulfide	298.053/24.42	H(+)		0.93 (0.19)	1.98 (0.95)**
L-Cystine	241.0314/24.1	H(+)	R(+)	0.95 (0.11)	1.6 (0.7)**
N1,N12-Diacetylspermine	287.2446/25.22	H(+)		1.1 (0.35)	2.22 (0.86)**
N1-acetylspermidine	188.1763/25.51	H(+)		1.09 (0.27)	2.34 (1.23)**
Pantothenic Acid	218.103/1.64	R(-)		0.87 (0.07)	1.37 (0.27)**
Phenylethylamine	105.034/2.73	H(+)		0.72 (0.2)	1.35 (0.42)**

S-(Hydroxymethyl)glutathione	336.0704/19.16	H(-)	R(-)	0.79 (0.29)	1.58 (0.32)**
S-Adenosylmethionine	399.1448/24.54	H(+)		0.98 (0.18)	1.99 (0.71)**
2,4-Diamino-butyric acid	117.0199/1.01	R(-)		0.79 (0.2)	2.7 (1.65)**
2,6-Diamino-heptanedioic acid	189.0404/0.96	R(-)		0.93 (0.17)	1.67 (0.71)**
4-(2-Aminophenyl)-2,4-dioxobutanoic acid	206.0457/4.62	R(-)	R(+), H(+)	1.03 (0.25)	2.13 (0.71)**
Glutarylcarnitine	276.144/16.1	H(+)		0.87 (0.8)	6.6 (4.27)**
3-indolecarboxylic acid glucuronide	338.087/19	H(+)		0.8 (0.37)	2.33 (1.21)**
4'-Methyl-(-)-epigallocatechin 7-glucuronide	495.1146/6.24	H(-)		0.84 (0.25)	2.65 (1.71)**
p-Cresol Glucuronide	283.0821/5.93	R(-)	H(-), R(+)	0.86 (0.23)	2.22 (0.71)**
Phenethylamine glucoronide, [M+FA-H]-	342.1186/1.07	R(-)		1.16 (0.14)	3.59 (1.93)*
N-butyrylglycine	144.0663/2.91	H(-)		0.69 (0.22)	2.48 (3.09)**
Phenylacetylgycine	192.0668/5.47	R(-)	H(+)	0.83 (0.15)	2.09 (0.64)**
Hippuric Acid (Benzoylglycine)	178.0513/4.29	R(-)	R(+), H(+)	0.75 (0.11)	1.78 (0.44)**
Adrenic acid	331.2628/14.04	R(-)		1.24 (0.33)	0.6 (0.23)**
Eicosadienoic acid	307.2626/14.44	R(-)		1.21 (0.56)	0.45 (0.25)**
LPC(17:0)	510.3554/11.18	R(+)		1.02 (0.24)	0.61 (0.11)**
LPC(20:3)	546.3555/10.46	R(+)		1.06 (0.14)	0.49 (0.19)**
LPC(22:4)	572.3714/10.93	R(+)		1.09 (0.11)	0.46 (0.13)**
LPC(22:5)	570.3558/10.2	R(+)	R(-)	1.09 (0.15)	0.44 (0.14)**
LPC(O-18:1)	508.3754/15.44	H(+)		1.01 (0.3)	0.44 (0.12)**
LPC(P-16:0)	480.344/14.86	H(+)		1.01 (0.09)	0.44 (0.11)**
PA(15:1)	375.1824/1.24	H(-)		0.95 (0.11)	0.53 (0.12)**
PA(29:2)	603.2906/12.67	H(+)		1.06 (0.19)	0.51 (0.28)*
PA(18:0), [M+Cl]-	473.1437/6.69	R(-)		0.91 (0.3)	1.9 (0.46)**
PA(18:1), [M+Na]+	459.1281/11.73	H(+)		0.99 (0.43)	2.73 (1.22)**
PA(18:4)	431.096/13.22	H(+)		0.67 (0.33)	2.51 (2.02)**
PA(19:3)	445.3291/1.21	H(-)		1.19 (0.23)	0.57 (0.12)**
PA(20:4)	459.2496/11.45	H(+)		1.18 (0.57)	0.62 (0.17)*
Lyso PAF C-16, [M+Cl]-	516.3184/15.76	H(-)		0.94 (0.07)	0.57 (0.2)**
Choline	104.108/13.5	H(+)		0.77 (0.17)	1.41 (0.39)**
PC(14:0)	468.308/15.42	H(+)		1 (0.09)	0.51 (0.12)**
PC(15:0)	480.3082/10.42	R(-)	H(+)	1.19 (0.26)	0.65 (0.18)**
PC(16:0)	496.3407/15.25	H(+)	R(+)	0.96 (0.08)	0.58 (0.08)**
PC(36:5)	780.5517/13.2	H(+)		1.35 (0.59)	0.61 (0.27)*
PC(21:0)	608.3173/10.42	R(-)		1.08 (0.29)	0.59 (0.18)**
PC(16:1)	494.3232/15.29	H(+)	R(+)	0.99 (0.08)	0.57 (0.1)**
PC(17:0)	510.3541/15.17	H(+)		0.94 (0.06)	0.61 (0.11)**
PC(26:0)	650.3458/14.92	H(+)		1.12 (0.18)	0.56 (0.14)**
PC(17:1)	508.3381/15.19	H(+)	R(-)	1.14 (0.35)	0.72 (0.17)**
PC(18:0)	524.3723/12.06	R(+)		1.1 (0.26)	0.55 (0.12)**
PC(20:0)	564.3302/9.92	R(-)		1.02 (0.21)	0.5 (0.22)**
PC(32:1)	590.3451/10.45	R(-)		1.11 (0.12)	0.52 (0.22)**
PC(18:1)	522.3547/15.11	H(+)	R(+), R(-)	0.99 (0.05)	0.6 (0.1)**
PC(18:2)	520.3398/15.19	H(+)	R(+)	0.96 (0.05)	0.57 (0.1)**
PC(18:3)	518.3229/15.25	H(+)		1 (0.08)	0.59 (0.11)**
PC(20:1)	550.3841/14.98	H(+)		0.96 (0.16)	0.49 (0.14)**
PC(20:2)	548.3686/15.04	H(+)	R(+)	0.94 (0.16)	0.54 (0.15)**
PC(20:4)	544.3394/15	H(+)	R(+)	1 (0.03)	0.55 (0.13)**
PC(20:5)	542.3221/15.09	H(+)	R(+)	1.01 (0.11)	0.59 (0.13)**
PC(22:4), [M+FA-H]-	616.3612/10.95	R(-)		0.99 (0.19)	0.41 (0.14)**
PC(22:6)	568.3386/14.95	H(+)	R(+), R(-)	1.06 (0.05)	0.46 (0.13)**
PC(O-15:0/O-1:0)	482.3605/11.15	R(+)		1.05 (0.21)	0.62 (0.09)**
PC(O-16:0/0:0)	482.3602/15.58	H(+)		1.01 (0.17)	0.47 (0.09)**

PC(O-18:0/20:4)	796.6183/13.27	H(+)		1.26 (0.41)	0.56 (0.4)*
SN-Glycero-3-phosphocholine	258.1111/23.56	H(+)		0.93 (0.19)	0.4 (0.14)**
PE(18:2)	478.2917/12.63	H(+)		1.17 (0.23)	0.64 (0.16)**
PE(20:4)	502.2919/12.37	H(+)	R(-)	1.38 (0.16)	0.67 (0.11)**
PE(22:4)	528.3087/9.91	R(-)		1.08 (0.18)	0.59 (0.2)**
PE(22:6)	526.2919/12.35	H(+)		1.28 (0.16)	0.81 (0.23)**
PE(O-18:1)	466.3281/12.17	H(+)		1.05 (0.28)	0.62 (0.15)**
PE(P-16:0)	438.2968/12.26	H(+)	R(+)	1.06 (0.21)	0.64 (0.14)**
PG(20:2)	537.1672/11.2	H(+)		1.46 (0.91)	4.73 (3.93)*
PI(17:1)	585.2701/1.2	H(+)		0.83 (0.21)	0.56 (0.12)*
PI(18:0)	599.3195/10.1	R(-)		0.98 (0.22)	0.56 (0.23)*
PI(20:4)	619.2847/12.81	H(-)		0.87 (0.2)	0.38 (0.16)**
PI(P-18:0)	585.2702/15.11	R(+)		0.93 (0.12)	0.49 (0.1)**
PS(38:6)	806.502/10.42	R(-)		1.27 (0.28)	0.61 (0.3)**
PS(18:2)	520.264/13.78	H(-)		1.23 (0.27)	0.62 (0.27)**
PS(19:0)	538.3138/9.53	R(-)		1.01 (0.04)	0.63 (0.19)**
PS(22:6)	568.3612/12.08	R(-)		1.08 (0.25)	0.56 (0.17)**
PS(20:0)	566.3459/10.78	R(-)		1 (0.16)	0.51 (0.19)**
Cer(d18:0/16:0)	540.3653/15.64	H(+)		1.28 (0.41)	0.56 (0.17)**
CerP(d18:1/14:0)	588.3301/9.9	R(-)		1.04 (0.16)	0.53 (0.26)**
NeuAcα2-3Galβ-Cer(d18:1/16:0)	991.6709/15.23	H(+)		1.14 (0.47)	0.25 (0.08)**
C16 Sphingosine-1-phosphate	350.1488/12.72	H(-)		0.66 (0.16)	0.31 (0.11)**
Psychosine Sulfate	540.33/10.42	R(-)		1.06 (0.21)	0.55 (0.16)**
SM(d16:1/18:1)	701.5579/14.48	H(+)		1.37 (0.84)	0.66 (0.23)*
SM(d18:1/14:0)	675.5417/14.55	H(+)		1.41 (0.93)	0.73 (0.33)*
1-Methylguanosine	298.114/1.24	R(+)	H(+)	1.13 (0.09)	1.54 (0.38)**
1-methylnicotinamide	137.071/14.26	H(+)		0.93 (0.06)	3.89 (5.68)**
1-methyluric Acid	181.0359/10.41	H(-)		0.99 (0.33)	2.25 (0.67)**
3-Methylguanine	166.0717/9.63	H(+)		0.94 (0.18)	1.8 (0.64)**
3'-O-Methyladenosine	282.1196/17.53	H(+)		0.99 (0.23)	1.46 (0.37)**
3'-O-Methylguanosine	298.114/11.76	H(+)		0.97 (0.18)	1.66 (0.61)**
7-Methylguanine	166.0719/7.73	H(+)		0.87 (0.21)	1.55 (0.59)**
N2-Methylguanine	166.072/11.76	H(+)		0.93 (0.13)	1.83 (0.82)**
Ascorbic Acid	175.0249/0.79	R(-)		1.01 (0.47)	4.72 (3.46)**
7-alpha-Hydroxy-3-oxo-4-cholestenoate	431.3158/10.35	R(+)		0.96 (0.09)	0.64 (0.18)**
9,12,15-Octadecatrien-1-ol	265.2523/14.18	R(+)		1.3 (0.34)	0.71 (0.22)*
Galactosamine-1-phosphate	260.0742/23.31	H(+)		1.12 (0.21)	2.37 (0.86)*
Isobuteine	206.0456/4.8	H(-)		1.03 (0.16)	1.8 (0.56)**
N1-(5-Phospho-a-D-ribosyl)-5,6-dimethylbenzimidazole	357.0818/1.83	R(-)		0.98 (0.31)	1.94 (0.49)**
Taurochenodeoxycholic acid 7-sulfate	580.4305/14.83	H(+)		1.25 (0.35)	0.52 (0.19)**
Aspartyl-aspartate	249.0753/5.94	R(+)		0.93 (0.12)	1.91 (0.69)**
Glutaminyl-Serine, [M+H-H2O]+	216.0974/17.09	H(+)		1.37 (1.15)	0.29 (0.26)*
Hydroxyprolyl-glutamine	258.108/23.73	H(-)		1.35 (0.68)	0.5 (0.15)**
Methionyl-Glutamate, [M+HAc-H]-	336.1388/23.43	H(-)		0.87 (0.31)	3.23 (1.73)**
Phenylalanine-Tryptophan	352.1644/12.64	H(+)		0.88 (0.23)	0.46 (0.13)**
Phenylalanyl-Glutamine	292.0808/12.23	H(-)		0.97 (0.18)	1.98 (0.79)**
Serinyl-Methionine	235.082/1.34	R(-)		1.67 (0.38)	3.75 (1.26)**
6,8-Dihydroxypurine	153.0402/12.22	H(+)		1.01 (0.15)	2.94 (1.18)**
Adenosine	250.0928/1.94	H(+)		0.87 (0.32)	2.71 (2.1)*
Deoxyguanosine	266.0878/12.32	H(-)		1.43 (0.67)	6.43 (3.29)**
Guanosine	282.0829/14.25	H(-)	R(+), R(-)	1.22 (0.5)	3.73 (2.09)**
Hypoxanthine	137.0462/11.17	H(-)	H(+)	1 (0.28)	3.08 (1.99)**
Inosine	269.0876/11.18	H(+)	R(+), R(-)	1.38 (0.62)	3.77 (2.24)**

Succinoadenosine	382.0993/1.79	R(-)		1.03 (0.15)	2.09 (0.61)**
Uric Acid	169.0348/14.69	H(+)	R(-)	0.72 (0.32)	5.43 (4.78)**
Xanthosine	285.0818/12.17	H(+)	R(-)	1 (0.35)	2.65 (0.95)**
Cytosine	112.0505/9.76	H(+)		0.61 (0.16)	1.66 (0.52)**
5-hydroxymethyldeoxycytidylic acid	320.0615/3.36	H(+)		0.77 (0.22)	2.87 (2.2)*
Cytidine, [M+H-H2O]+	226.0817/9.78	H(+)		0.94 (0.14)	1.72 (0.49)**
Cytidine Monophosphate	322.2006/1.33	H(-)		0.97 (0.16)	0.54 (0.16)**
Thymidine	241.0824/1.38	R(-)		1 (0.17)	1.82 (0.43)**
Thymine	127.05/1.77	H(+)		1.1 (0.21)	1.64 (0.34)*
Uridine	243.0618/0.92	R(-)		0.81 (0.19)	2.07 (0.73)**

[a] Significant based on Wilcoxon-Mann-Whitney significance test of fold change values for Sham vs Trauma: *$p < 0.05$ **$p < 0.01$.

Table S4. Top metabolic pathways affected after the trauma injury.

	Total Cmpd	Hits	Raw p	-log(p)	Holm adjust	FDR	Impact
**Glycerophospholipid metabolism**	36	8	5.274E-07	6.278	1.002E-05	5.274E-06	0.137
**Purine metabolism**	65	7	0.005	2.300	0.033	0.007	0.025
**Sphingolipid metabolism**	21	5	1.565E-06	5.806	2.816E-05	1.043E-05	0.294
**Pyrimidine metabolism**	39	4	0.001	3.267	0.006	0.001	0.117
**Cysteine and methionine metabolism**	33	3	0.033	1.484	0.090	0.035	0.157
**Arginine biosynthesis**	14	2	7.473E-04	3.127	0.008	0.001	0.305
**Phenylalanine metabolism**	10	2	4.405E-04	3.356	0.006	0.001	0.238
**Arginine and proline metabolism**	38	2	0.002	2.728	0.017	0.003	0.058
**Primary bile acid biosynthesis**	46	2	7.422E-05	4.130	0.001	0.000	0.051
**Ether lipid metabolism**	20	2	4.683E-08	7.330	9.365E-07	9.37E-07	0.000
**Aminoacyl-tRNA biosynthesis**	48	2	0.001	2.960	0.011	0.002	0.000

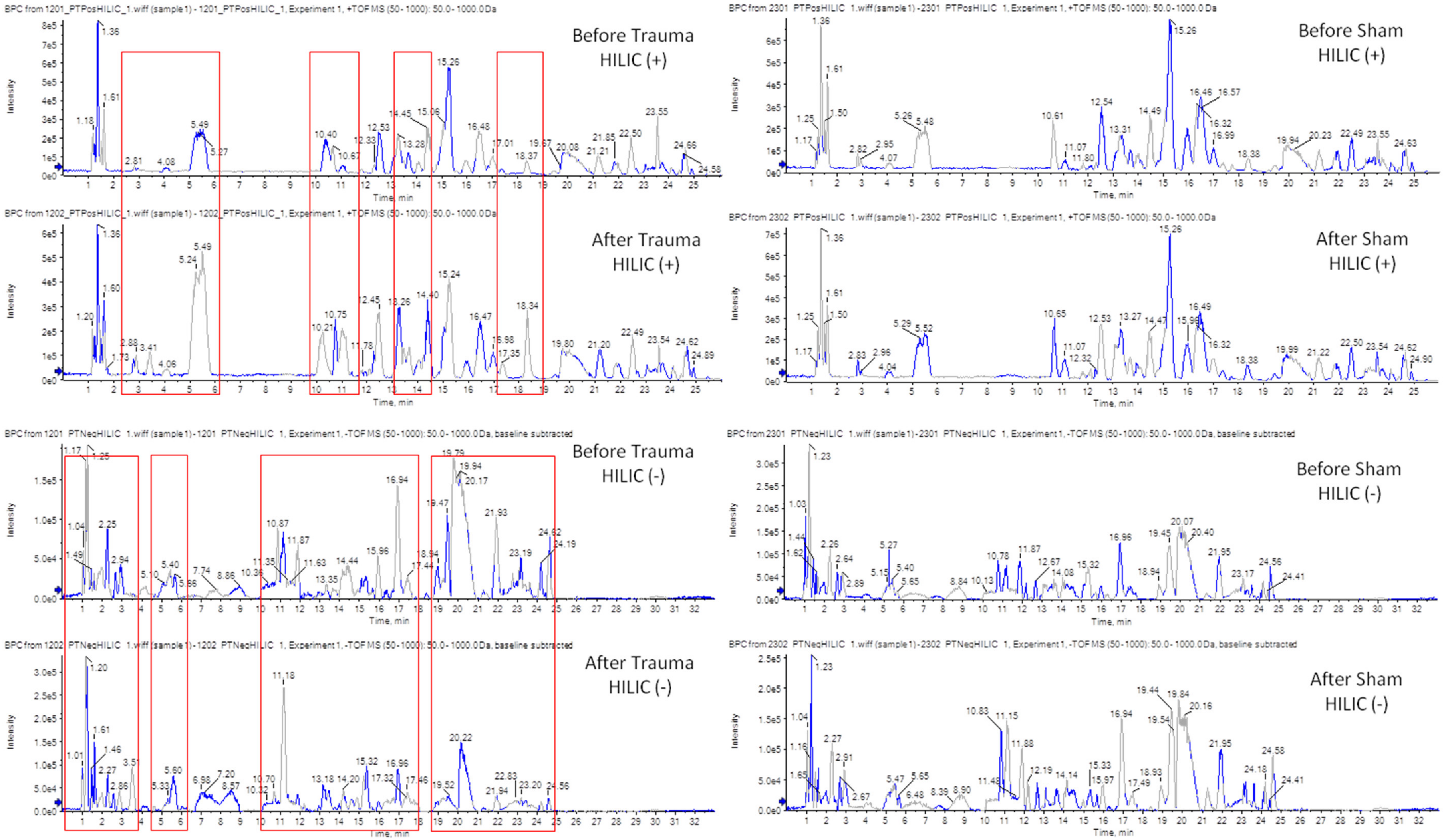

Figure S1. Comparison of base peak chromatograms of the HILIC LC-MS analyses for the sham and trauma samples.

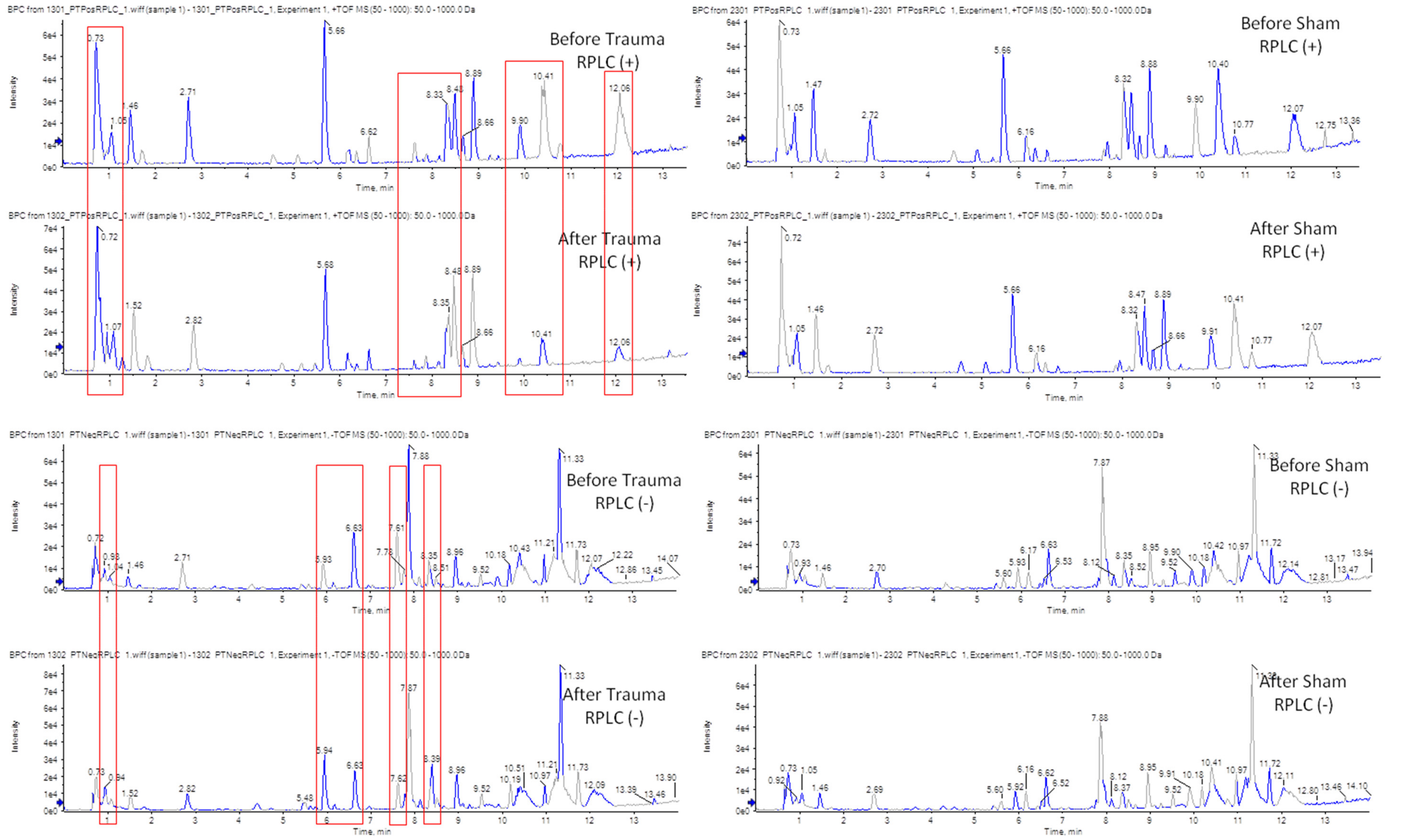

Figure S2. Comparison of BPCs of the RP LC-MS analyses for the sham and trauma samples.

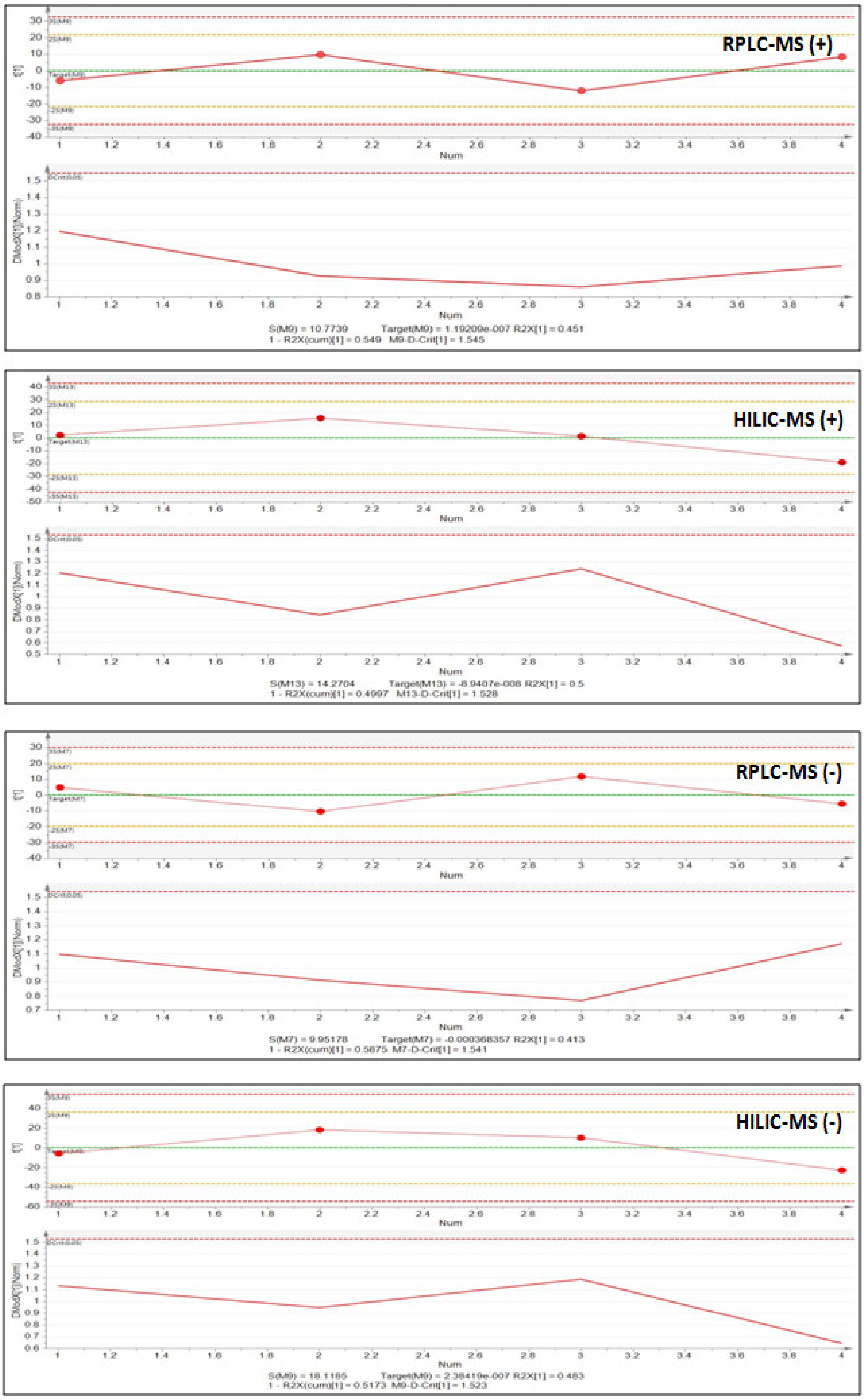

Figure S3. Shewhart control charts of the LC-MS pooled QC samples.
